# Towards polycotton waste valorisation: depolymerisation of cotton to glucose with polyester preservation†

**DOI:** 10.1039/d5su00230c

**Published:** 2025-07-24

**Authors:** Nienke Leenders, Gerard P. M. van Klink, Gert-Jan M. Gruter

**Affiliations:** a Industrial Sustainable Chemistry Group, Van't Hoff Institute for Molecular Sciences, University of Amsterdam Science Park 904, 1098 XH Amsterdam The Netherlands g.j.m.gruter@uva.nl; b Avantium Support BV Zekeringstraat 29 1014 BV Amsterdam The Netherlands

## Abstract

Every second, the equivalent of a garbage truck filled with textile waste is discarded. Due to the complex composition of clothing materials, more than 80 wt% of this waste is either incinerated or sent to landfills. Currently, only 15 wt% of textiles are recycled, and of that fraction, approximately 93 wt% undergoes downcycling, resulting in lower-value products. Generally, about 50 wt% of textile waste is composed of polycotton—a blend of cotton, a glucose-based polysaccharide, and polyester, primarily poly(ethylene terephthalate) (PET). Although this combination leverages the advantageous properties of both fibres, full valorisation of these materials is complex due to their blended structure. Simultaneously, there is an urgent need to transition away from fossil-based feedstocks. Cotton-rich textiles present a promising alternative as a non-food-based glucose source for the chemical industry, owing to their high cellulose content and widespread availability. Therefore, this review explores the current state-of-the-art methods for hydrolysing cotton into glucose through acid and/or enzymatic hydrolysis, while preserving the polyester component. These techniques enable the effective separation and subsequent valorisation of both cotton and PET fractions, facilitating their reuse in the production of new materials.

## Abstract 2

Polycotton waste textile valorisation; upgrading waste cotton from polycotton (left) to the platform chemical glucose without affecting the polyester (right).

## Graphical abstract

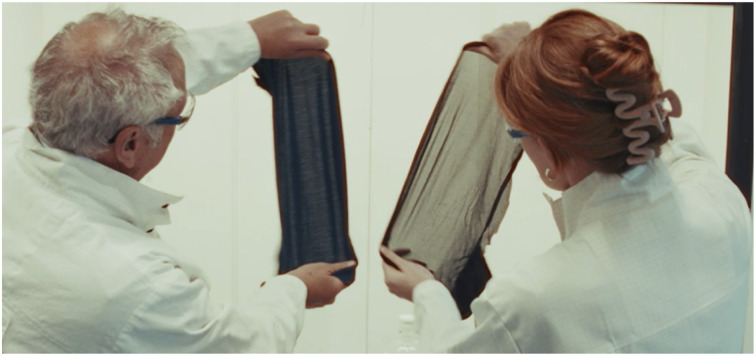

**Sustainability spotlight**

With a recycling rate of less than 1%, the textile industry is the third most polluting industry, directly after the oil and gas industry, and agriculture industry. The complex nature of textile materials poses a formidable challenge for true recycling. It is imperative for the industry to transition towards circular and low-carbon practices, especially in light of the Extended Producer Responsibility (EPR), which makes the textile producers financially and/or physically responsible for their post-consumer waste. Polycotton is the largest volume textile waste and currently is not recycled in a cost competitive way, but new technology options are under development. Circularity for polycotton waste addresses UN sustainability goals 9, 11, 12 and 13.

## 1 Introduction

Today, the consumer buys 60% more items compared to 15 years ago and the average lifetime of an item has decreased by 50%, resulting in the world-wide disposal of one garbage truck full of textile waste every second.^1,2^ In 2024, 92 million tonnes of textile waste was produced globally.^3^ Currently, only 15 wt% of post-consumer textile waste is recycled, with 93 wt% of this being downcycled to low-value application and less than 1 wt% being closed-loop recycled (to the same or similar quality application(s)).^2^ The other 85 wt% of the textile waste is landfilled or incinerated. The low textile recycling rate is caused by the complexity of the material, as textile is often a blend of different fibres, and contains multiple components such as zippers, seams, buttons, labels and prints.^4–7^ As a result, mechanical recycling is currently the best option for blended/mixed textiles. However, as mechanical recycling leads to a decrease in fibre properties/quality, there is a growing interest in chemical recycling technologies.^8,9^

Meanwhile, the goal is to move away from fossil resources and find sustainable alternatives for the production of chemicals and their derived products.^10^ Sustainable alternatives include the use of bio-based materials, waste valorisation, green energy and carbon capture utilisation. Textile waste is especially interesting as cotton, the second most used fibre in clothing, is a polysaccharide of glucose.^11^ This means that cotton-containing textile waste could be a sugar source for the chemical industry that does not originate from edible biomass such as starch, thus avoiding a possible future conflict with the food industry when significant quantities of fossil-based feedstocks are substituted with bio-based feedstocks. Lignocellulosic waste such as agro- and forestry residues can also be an alternative glucose source, however, the typical cellulose content is lower in lignocellulosic waste.^12^ Additionally, besides cellulose, lignocellulosic waste also includes hemicellulose, which would lead to a mixture of hexose and pentose sugars resulting in the need of more downstream processing for most applications. Thus, textile waste seems to be an interesting candidate as a source for second-generation glucose.

About 50 wt% of the textile waste is a blend of cotton and polyester, named polycotton.^11,13^ This blend combines the durability of the polyester with the breathability and comfort wear of cotton. Techniques for polycotton recycling can be classified into four categories, namely cellulose dissolution, cellulose depolymerisation, polyester dissolution and polyester depolymerisation.^4^ For full recycling of polycotton textile waste, cellulose dissolution or depolymerisation is combined with polyester dissolution or depolymerisation. For glucose production, cellulose depolymerisation methods need to be utilised, which includes acid and enzymatic hydrolysis as well as hydrothermal treatment. Besides glucose, the products of these methods can also include microcrystalline cellulose, cellulose nanocrystals and cellulose powder obtained *via* partial hydrolysis. Since polyester and cotton have different chemical properties, the polyester is usually not affected during dissolution and hydrolysis of cellulose, also known as cellulolysis.^14^ In theory, this would allow for a full separation of cotton and polyester. When cellulolysis is done properly, an (oligomeric) sugar solution and solid polyester fibres can be obtained. The sugar solution can be used (preferably after acid removal) for sequential production of value added chemicals such as bioethanol, biohydrogen, succinic acid, glucaric acid, lactic acid, sorbitol and 5-(chloromethyl)furfural.^15–18^ To make the downstream processing as convenient as possible, polyester degradation should be avoided as its monomers (terephthalic acid (TPA) and ethylene glycol (EG)) can contaminate the sugar stream. Additionally, as in the current economy recycled polyester is seen as a valuable product, the residual polyester waste textile should be conserved so it can be further processed into recycled polyester.

Therefore, the focus of this review will be to present an overview of cellulolysis studies that produce glucose and residual polyester from polycotton materials, so both fractions can be conveniently valorised. The glucose, produced from waste textile, can be used as a feedstock for various chemical processes. Since it results from non-edible mass, it provides the chemical industry with a sugar supply that is not in competition with the food industry. Additionally, the polyester residue can be used by polyester recycling companies to produce recycled polyester, leading to a full valorisation process.

This review outlines the state of the art methods to chemically recycle cellulose to glucose, while preserving the polyester fraction. Information on the cotton fibre, with in-depth information on cellulose in cotton, the polyester fibre as well as the structure of the blend is provided. Then, an assessment is given of the state of the art cellulolysis methods that produce glucose and solid polyester remains. In addition, the sustainability of these methods is assessed by their use of energy for the cellulolysis process. Lastly, the viability of these recycling methods on larger scales is considered.

## 2 Polycotton, the most common blend

Polycotton, a blend of polyester and cotton, is a widely used and popular fabric in the textile industry. The ratio of polyester to cotton per item varies considerably but usually an item contains 50 to 85 wt% cotton and 15 to 50 wt% polyester. The polycotton blend combines the best qualities of both fibres, the strength and resistance to stretching and shrinking from polyester and the soft and breathable characteristics from cotton.

### 2.1 Cotton, the most used natural fibre

#### 2.1.1 The structure of a cotton fibre

**Fig. 1:**
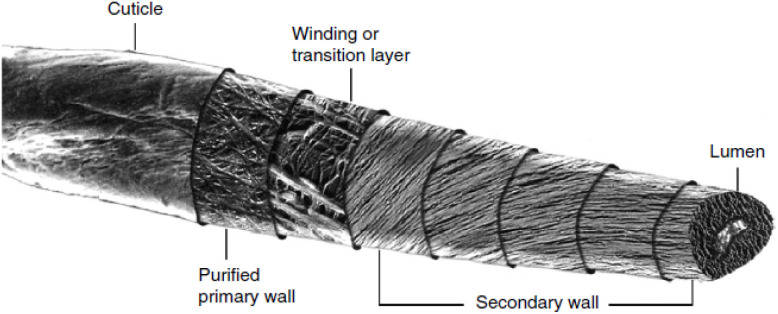
Structure of a cotton fibre produced *via* transmission electron microscopy and scanning electron microscopy images. Reproduced from Nam and Condon (2014).^23^ Copyright (2014) with permission from Springer Nature.

Cotton is the most used natural fibre and each cotton fibre is a single cell that grows from the surface (epidermal cell) of the seed coat in the cotton plant.^11,19,20^ The fibre develops in the cotton boll in four overlapping stages: initiation, elongation, secondary wall biosynthesis and maturation.^20–22^ The fibre consists of five components, arranged from the outside to the inside: the cuticle, primary wall, winding/transition layer, secondary wall, and lumen (Fig. 1).^23^ The cuticle and primary wall are made from an inner network of microfibrils, randomly organised within a mixture of waxes, pectins, proteins and other noncellulosic materials which protect the fibre.^24^ The microfibrils are produced by coalescence of the cellulose chains during the biosynthesis, where the glucan chains associate in a regular intermolecular arrangement.^24–26^ As a result of the large amount of intra- and intermolecular hydrogen bonds that can be formed within and between the cellulose chains (*vide infra*), the chains form a dense and highly crystalline network.^27,28^ The degree of crystallinity in a cotton fibre ranges between 70 and 80%.^29^ In these crystalline regions, the highly ordered cellulose chains are tightly packed and contain many hydrogen bonds leading to a low reactivity.^30^ In contrast to the amorphous regions, where there is more disorder and less hydrogen bonding, the cellulose chains are more susceptible to chemicals and enzymes.

The high crystalline order is not only a result of the intra- and intermolecular hydrogen bonding and the regular chemical structure, as the steric repulsion and attractive dispersion interaction of the chains can account for up to 70% of the cohesive energy of cellulose.^26,31^ Beneath the primary wall, the winding layer consists of bands of helical microfibrils that are laid down in a lacy network. The intermeshed fibrillar network of the primary wall and the woven mat of fibres of the winding layer provide a casing that limits swelling of the secondary wall. This causes the microfibrils to orientate more along the fibre axis. Additionally, the primary wall and winding layer protect the secondary wall against damage. The secondary wall consists of layers of nearly parallel fibrils. These densely packed fibril layers in the secondary wall are pure cellulose. The centre of the fibre, the lumen, contains the dried residues of the cell protoplasm, which is the only noncellulosic material in the fibre other than the cuticle and primary wall.

The fibre development stops when the boll opens, which results in the dehydration of the fibre. Since the primary wall is less able to shrink due to its rigid network structure, the fibre wrinkles and moulds the underlying fibre layers producing folds and twists resulting in a twisted kidney shaped fibre.^20^ After opening of the boll, the cotton is harvested and shipped to the fibre production plant. More information on the process of yarn and fabric production can be found in the ESI, Section 1.† After the yarn production, nearly all of the non-cellulosic components have been removed and the cellulose content in cotton is >99 wt%.^32,33^

#### 2.1.2 Cellulose in cotton

**Fig. 2:**
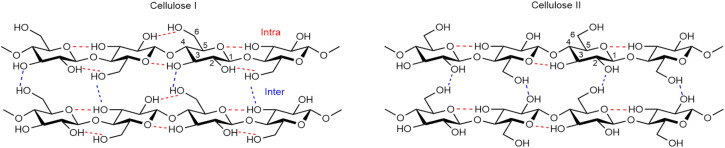
Intra- and intermolecular hydrogen bonding network in cellulose I and cellulose II.^38^

Cotton's main component is cellulose, which is synthesised by the enzyme complexes *via* condensation of glucose molecules.^22^ Cellulose is a linear polysaccharide of β-d-glucopyranose units that are linked *via* β-1,4-glycosidic bonds (Fig. 2). The β-d-glucopyranose unit, also known as an anhydroglucose unit (AGU), contains two secondary equatorial hydroxy groups positioned at C2 and C3 and one primary hydroxy group at C6 with this CH_2_OH side group in *trans*–*gauche* position relative to the O5–C5 and C4–C5 bonds.^34^ The hydroxy groups at C2 and C6 are the most accessible for reaction and functionalisation.^35^ Every second AGU is rotated 180° to provide the desired bond angle for the acetal oxygen bridge in beta position.^34^ The polysaccharide consists of a reducing (C1–OH) and a nonreducing end (C4–OH). The reducing end is more reactive since the cyclic hemiacetal is in equilibrium with the aldehyde. The closed ring structure of the nonreducing end leads to a low reactivity.^34^ Despite their high reactivity, the reducing ends are often ignored as they are present in small quantities in cellulose.

Cellulose can be found in many different crystalline forms, or polymorphs, depending on the source and subsequent treatment. Native cotton classifies as cellulose I (more specifically I_β_; see Fig. 2) and contains intramolecular O3–H–O5′ and O2–H–O6′, and intermolecular O6–H–O3′′ bonds. It is said that cellulose I is metastable, as regeneration of cellulose from a solution or upon alkaline treatment leads to a different polymorph, namely cellulose II (see Fig. 2).^26,27,36,37^ In cellulose II, the hydroxymethyl group has shifted from *trans*–*gauche* to a *gauche*–*trans* position and the cellulose chains are linked by intramolecular O3–H–O5′ and intermolecular O6–H–O2′′ bonds. Cellulose I and II are the most common polymorphs. The main difference between cellulose I and II is the direction of the chains. A parallel configuration is formed with cellulose I, where cellulose II has an antiparallel configuration, which is the lowest energy polymorph.^34,38^

Cellulose consists of a high amount of hydrogen bonds, which according to many publications is the reason for the low solubility of cellulose in water.^34,39–42^ The formed intra- and intermolecular hydrogen bonds would prevent the dissolution in water. However, some scientists reason that the insolubility of cellulose in water and other common solvents is due to the fact that cellulose is an amphiphilic polymer.^43–45^ These amphiphilic interactions are caused by hydrophobic and hydrophilic regions, where in the case of cellulose the equatorial hydroxy groups and the axial hydrogen atoms are linked to the hydrophilic and hydrophobic interactions, respectively.^44,46–48^ The axial hydrogen atoms would generate inter-sheet hydrophobic interactions along the glucopyranose rings disfavouring dissolution.^44,49–51^ These hydrophobic regions combined with the rigid structure of cellulose lower the entropy, resulting in a positive free energy.^44^ The free energy, which is the sum of the positive enthalpy and the negative temperature × entropy, should be negative for dissolution to occur.^45^ The enthalpy is depended on the intermolecular interactions, where more intermolecular interactions increase the enthalpy, and the entropy is affected by the molecular weight of the material. Higher molecular weight polymers have a lower entropy in solution compared to low molecular weight polymers, as larger molecules are more rigid and have fewer conformational states, leading to lower entropy and unfavouring dissolution.^45,52,53^ The effect of the chain length is also visible when considering glucose, cellobiose and oligomers of cellulose up to 10 repeating units, which are soluble in water as their entropy is much higher.^54^ Altogether, the amphiphilic characteristic of cellulose would be the result of the insolubility of cellulose in water. However, the scientific community is still in discussion and has not yet accepted one theory for the solution behaviour of cellulose.

### 2.2 Polyester, the most used synthetic fibre

**Fig. 3:**
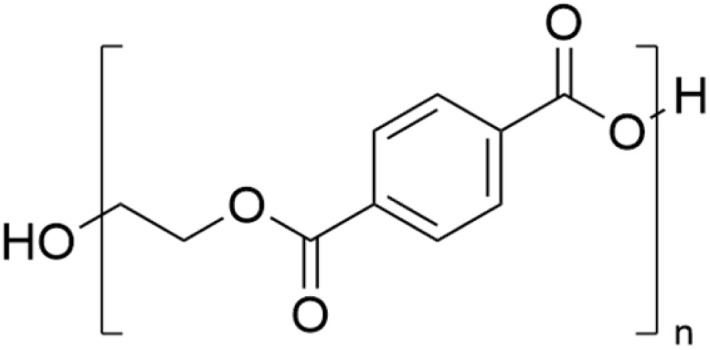
Chemical structure of poly(ethylene terephthalate).

Among all the fibres in the textile industry, polyester is the most used (synthetic) fiber.^11^ It is a filament fibre and synthetic thermoplastic polymer containing two ester groups in each repeating unit in the polymer chain. Within the polyester class, poly(ethylene terephthalate) (PET) (Fig. 3) is the most commonly used for fibre production due to its low moisture absorbency, excellent wear resistance, low price and high weather, light and abrasion resistance compared to other fibres. Additionally, the properties of the fibres can be easily adjusted, as the material can easily be modified to obtain the desired elasticity, pilling tendency, ability to dye and shrinkage properties.^55^ A brief overview of the synthesis of PET and the production of PET fibres can be found in the ESI, Section 2.†

### 2.3 Polycotton fabrics

**Fig. 4:**
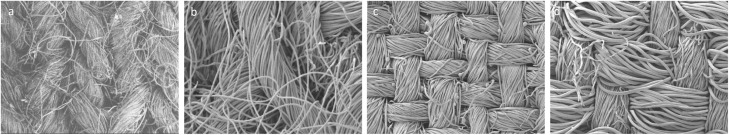
Scanning electron microscopy images of several polycotton fabrics. (a) Of knitted fabric of blended yarn (56 polyester/44 cotton),^57^ (b) knitted fabric of 100% polyester yarn and 100% cotton yarn (17 polyester/83 cotton),^58^ (c) woven fabric of cotton yarn warp and blended yarn weft (25 polyester/75 cotton),^58^ (d) woven fabric of polyester yarn warp and cotton yarn weft (30 polyester/70 cotton).^58^

Polycotton fabrics can be produced by blending polyester and cotton into one composite yarn during spinning or by knitting or weaving with a pure cotton and a pure polyester yarn.^56^ Leenders *et al.* and Machnowski and Was-Gubala took scanning electron microscopy images of knitted fabric of composite yarn (56 polyester/44 cotton) (Fig. 4a),^57^ knitted fabric of 100% polyester yarn and 100% cotton yarn (17 polyester/83 cotton) (Fig. 4b),^58^ woven fabric of cotton yarn warp and composite yarn weft (25 polyester/75 cotton) (Fig. 4c),^58^ and woven fabric of polyester yarn warp and cotton yarn weft (30 polyester/70 cotton) (Fig. 4d).^58^ The structure of the composite yarn depends on the spinning process used. More information on this can be found in the work of Chen and coworkers.^59^

## 3 Cellulolysis, the hydrolysis of cellulose to glucose

The solubilisation and depolymerisation of cotton to glucose (and glucose oligomers), can be achieved *via* acid hydrolysis, enzymatic hydrolysis and hydrothermal treatment. As acid hydrolysis with a low acid concentration at high temperature is basically the same process as hydrothermal treatment, the depolymerisation methods will be categorised in dilute acid hydrolysis, concentrated acid hydrolysis and enzymatic hydrolysis.

Here, we classify dilute acid hydrolysis as a process where the acid concentration ranges between 0.5—10 wt% with a reaction temperature above 50 °C. The concentrated acid hydrolysis is defined as a process with an acid concentration above 10 wt% and a reaction temperature below 50 °C.

Both acid and enzymatic hydrolysis can selectively depolymerise cotton without depolymerising the polyester fraction. These approaches provide waste textile recycling and when done properly, can lead to full valorisation of both the cotton and polyester fraction. Additionally, these methods allow for the valorisation of low-quality fibres which would otherwise be downgraded or even destructed by incineration.

For full polycotton valorisation, it is essential to completely separate both fractions. As studies on acid hydrolysis of cotton to glucose from polycotton materials are limited, we have also included studies that hydrolyse (acidic and enzymatic) 100% cotton materials in this review to provide a complete overview and their suitability for polycotton materials is discussed. In all studies, the starting material is first separated from nonfabric materials such as zippers, buttons and labels, and then subjected to size reduction such as cutting.

### 3.1 Acid hydrolysis

Due to the different chemical nature of polyester and cotton, polyester stays unaffected under certain conditions in an acidic environment (*vide infra*) whereas cotton is, in principle, able to fully depolymerise into soluble glucose and oligomers, which, in theory, leads to complete separation of the two components. Acid hydrolysis of cellulose can be performed with organic and inorganic acids at varying concentrations and temperatures. Table 1 presents the state of the art processes for acid hydrolysis of (poly)cotton materials.

**Table 1:** Acid hydrolysis for the depolymerisation of cotton to glucose

Entry	Acid	PET/cotton ratio	Textile	Pretreatment	Conditions acid hydrolysis							References
					Acid conc. [wt%]	Solid (cotton) loading [g L^−1^]	Temp. [°C]	Reaction time [h]	Molar glucose yield [%]	Suitable for polycotton valorisation	Energy consumption [Wh]	
1	H_2_SO_4_	0/100	Bed linen	H_2_SO_4_ (80 wt%), solid loading 5 wt%, 30 °C, 1 h	5	1.8	121	1	84	No	116	Sanchis-Sebastiá *et al.*, 2021 (ref. 67)
2	H_2_SO_4_	0/100	Bed linen	H_2_SO_4_ (80 wt%), solid loading 75 wt%, 30 °C, 1 h	10	50	100	1	72	No	91	Ruuth *et al.*, 2022 (ref. 6)
3	H_2_SO_4_	0/100	Cotton	—	55	40	40	1.5	66.5^a^	No	12	Chu *et al.*, 2011 (ref. 136)
4	H_2_SO_4_	0/100	Defatted cotton fibre	H_3_PO_4_ (85 wt%), solid loading 5 wt%, 50 °C, 1 h	0.5	0.2	180	0.5	43	No	192	Amiri and Karimi, 2013 (ref. 73)
5	H_2_SO_4_	0/100	Yarn	—	2	50	160	2	6.5	No	159	Binczarski *et al.*, 2021 (ref. 74)
6	H_3_PO_4_	0/100	Cotton fibre	—	2	33	140	2	38.8	Yes	136	Binczarski *et al.*, 2022 (ref. 75)
7	H_3_PO_4_	50/50	Fabrics	—	2	30 (15)	140	2	69	Yes	136	Binczarski *et al.*, 2024 (ref. 65)
8	HCl	35/65	Blue fabric	—	1.5	N.D. (N.D.)	150	3	15.5	Yes	148	Hou *et al.*, 2018 (ref. 14)
9	HCl	56/44	Blue post consumer waste	—	43	50 (22)	RT	18	80	Yes	0	Leenders *et al.*, 2025 (ref. 57)
10	HCl/formic acid	0/100	Degreased cotton	—	4/78	40	65	5	22.6	Yes	30	Sun *et al.*, 2007 (ref. 69)
11	Citric acid	75/25	Blue fabric	—	1	10 (2.5)	225	0.83	22	No	233	Kawamura *et al.*, 2020 (ref. 66)

a Reducing sugar yield.

#### 3.1.1 Acid hydrolysis of polyester

Although acid hydrolysis is commonly used for cotton hydrolysis, it can also be used for PET hydrolysis, under certain conditions. Acid hydrolysis of PET produces TPA and EG and has been studied by Yoshioka *et al.*,^60^ Mancini and Zanin,^61^ De Carvalho *et al.*,^62^ Ohmura *et al.*,^63^ and Ikenaga *et al.*^64^

From their work, it could be concluded that a lower concentration sulfuric acid (37 wt% (4.8 M)), phosphoric acid (1.75, 3.5 and 85 wt% (0.2, 0.4, 14.7 M, respectively)) and hydrochloric acid (1 wt% (0.3 M)) were able to hydrolyse PET into TPA and EG when the reaction is performed at elevated temperature and pressure. High concentration of sulfuric acid (96 wt% (18 M)) also hydrolysed PET at room temperature. A high concentration of HCl (43 wt% (14.3 M)) at room temperature did however not lead to polyester hydrolysis.^57^ Therefore, to be able to valorise both the cotton and polyester fraction, it is important that the type of acid and the reaction conditions are selected in such a way that only the cotton is hydrolysed and the polyester remains intact.

When assessing the studies mentioned in Table 1 on their ability to only hydrolyse cotton to glucose while leaving PET unhydrolysed, only five studies were suitable. All the studies using sulfuric acid (Table 1, entries 1–5) were expected to hydrolyse cotton and polyester. This could also explain why no literature could be found on the acid hydrolysis of polycotton materials with sulfuric acid, solely on 100% cotton materials. Binczarski *et al.* specifically mentioned that during the acid hydrolysis of polycotton with H_3_PO_4_ ‘no compounds characteristic of synthetic fibre depolymerisation were detected in the hydrolysate’,^65^ indicating that the processes using 2 wt% H_3_PO_4_ (0.2 M) at 140 °C for 2 h (Table 1, entries 6 and 7) can be used to depolymerise cotton to glucose without PET destruction. Additionally, Hou *et al.* were able to recover >96 wt% of the PET after hydrolysing the cotton to glucose, making this technology (Table 1, entry 8) also suitable for polycotton valorisation. Leenders *et al.* was also able to fully recover PET after acid hydrolysis of polycotton material with 43 wt% HCl.^57^ It is also expected that the hydrolysis performed by Sun *et al.* will be suitable for polycotton valorisation, as they perform the reaction with 4 wt% HCl (1.1 M)/78 wt% formic acid at 65 °C, since the acid concentration and/or reaction temperature is too low. Kawamura *et al.* mentioned that they found solid residue after treating polycotton material with 1 wt% citric acid (0.1 M) after 50 min at 225 °C.^66^ However, they did not mention whether they were able to fully recover the PET. Since Ikenaga *et al.* showed that after 30 minutes at 227 °C and 2.6 MPa with a microwave assisted neutral hydrolysis, a 76% TPA yield was obtained,^64^ we wonder whether full recovery of PET was obtained. Therefore, more information on the recovery rate of PET during this hydrolysis would be conclusive.

To conclude, the studies of Binczarski *et al.* (Table 1, entries 6 and 7), Hou *et al.* (Table 1, entry 8), Leenders *et al.* (Table 1, entry 9) and Sun *et al.* (Table 1, entry 10) are suitable for polycotton valorisation *via* cellulolysis of cotton and polyester preservation.

#### 3.1.2 Dilute *vs.* concentrated acid

Acid hydrolysis can be categorised as either dilute acid hydrolysis or concentrated acid hydrolysis. The dilute acid hydrolysis is often performed at high temperature and *vice versa*. Nine out of eleven studies used dilute acid hydrolysis at elevated temperatures. Low acid concentrations are often preferred over high acid concentrations as the decomposition rate of glucose to hydroxymethylfurfural (HMF), levulinic acid, formic acid and humins is lower.^67^ However, for the hydrolysis to be successful, a low acid concentration needs to be combined with a high temperature, which leads to a concomitant autogenous pressure build-up and a higher decomposition rate of the formed glucose.^68,69^

From all eleven studies considered, six studies perform a dilute acid hydrolysis without pretreatment which results in glucose yields ranging from 6.5–69%. Three studies consider the dilute acid hydrolysis with pretreatment with glucose yields ranging from 43–84%, with 84% glucose yield being the highest reported for acid hydrolysis (Table 1, entry 1).^67^ Only two studies used concentrated acid hydrolysis, both without pretreatment (Table 1, entries 3 and 9), which obtained glucose yields of 22.6 and 80%, respectively.

A method to compare the effectiveness of the dilute and concentrated acid hydrolysis with each another is by calculating the combined severity factor (CSF). The CSF combines the reaction time, reaction temperature and acid concentration into a singular factor and provides a manner to compare the effect of these factors on the glucose yield in various processes.CSF = log(*R*_0_) − pH
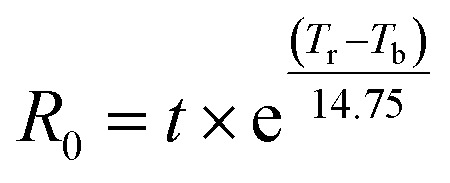
where *t* = reaction time (min), *T*_r_ = reaction temperature (°C), *T*_b_ = base temperature (100 °C), and constant 14.75 is the activation energy for a first order system.^70,71^

**Fig. 5:**
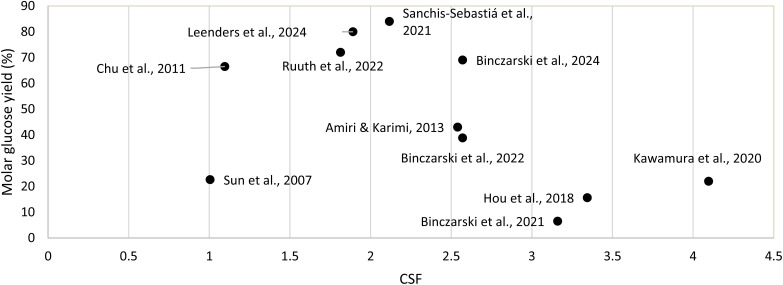
Combined severity factor (CSF) *vs.* glucose yield of the acid hydrolysis studies.

In Fig. 5, the calculated CSF of the mentioned studies performing acid hydrolysis on (poly)cotton materials are shown. An optimum appears to be around a CSF of 2.1, where the work of Sanchis-Sebastiá *et al.* hydrolysed 100% cotton bed linen with 5 wt% sulfuric acid (H_2_SO_4_) (0.5 M) at 121 °C for 1 h which yielded a 84% glucose yield (Table 1, entry 1). Maximum glucose yield is obtained when the CSF is sufficiently high to solubilise cellulose, which here seems to be around 2.1, yet not so high that it causes glucose degradation.^68,70^ The degradation of glucose is a possible explanation for the lower glucose yield of studies with a CSF beyond 2.1 (Fig. 5). However, a more in depth research would be needed to verify this conclusion, as the cotton loading has not been included in these calculations and it is known that the glucose yield is affected by the solid loading. Additionally, the effect of the pretreatment, which is known to have an impact on the solubilisation of cellulose, is also not included in the CSF. Nevertheless, using the CSF could help explain why some studies achieve higher glucose yields compared to others.

Additionally, in Table 1 it can be seen that sulfuric acid is a commonly used acid, as it was used in five (out of the eleven) studies. Sulfuric acid is often preferred due to its strong acidity and low volatility. Hydrochloric acid has a higher acidity, however, concentrations above 37 wt% HCl will lead to fuming HCl, making it less convenient to handle in the laboratory.

#### 3.1.3 Textile usage

Unfortunately, only four of the eleven studies performed acid hydrolysis on polycotton materials. As mentioned above, complete separation is important for the sufficient recycling of both fractions. Only in the studies of Hou *et al.* and Leenders *et al.*, cotton free polyester was obtained after acid hydrolysis.^14,57^ Binczarski was unable to fully remove cotton from the polycotton fabrics, as the IR spectrum still showed a band at 3331 cm^−1^, related to the OH stretching of cotton.^65^ However, this was not an objective in this study and a prolonged reaction time might tackle this problem. That said, a longer reaction time would increase the CSF (currently 2.5), moving it further from the optimum, leading to a lower monomeric glucose yield. In the study by Kawamura *et al.*, the quality of the residual polyester fabric was not specified (*vide supra*).^66^

#### 3.1.4 Pretreatment

In addition to combining low acid concentrations with high temperatures for the main hydrolysis, several research groups also include an acid pretreatment before hydrolysing cotton. The acidic pretreatment is performed with a high acid concentration at low temperature to provide good solubilisation.^67^ Sanchis-Sebastiá *et al.* used the combination of a pretreatment with 80 wt% H_2_SO_4_ (14.1 M) at 30 °C for 1 h followed by a hydrolysis with 5 wt% H_2_SO_4_ at 121 °C for 1 h.^67^ Ruuth *et al.* also performed a pretreatment with 80 wt% H_2_SO_4_ at 30 °C for 1 h followed with a hydrolysis with 10 wt% H_2_SO_4_ (1.1 M) at 100 °C for 1 h (Table 1, entry 2).^6^ These methods are almost similar to the method designed by the National Renewable Energy Laboratory for quantitative sugar analysis, where a pretreatment is done with 72 wt% H_2_SO_4_ (12 M) at 30 °C for 1 h with a solid loading of 0.1 g mL^−1^ followed by a hydrolysis with 4 wt% H_2_SO_4_ (0.4 M) at 121 °C for 1 h.^72^ Likewise, Amiri and Karimi combined a phosphoric acid (H_3_PO_4_) pretreatment with H_2_SO_4_ hydrolysis.^73^ These researchers were all able to obtain fair to relative high glucose yields when performing a pretreatment and acid hydrolysis, with yields of 84, 72 and 43%, respectively.

Binczarski *et al.* performed only a 2 wt% H_2_SO_4_ (0.2 M) hydrolysis without pretreatment leading to a glucose yield of 6.5% (Table 1, entry 5).^74^ Not all studies without pretreatment with low acid concentrations led to such low yields. In 2022 and 2024, Binczarski *et al.* used 2 wt% H_3_PO_4_ at 140 °C for 1 h to hydrolyse pure cotton and 50/50 polycotton fabrics with glucose yields of 38.8 and 69%, respectively (Table 1, entries 6 and 7).^65,75^ When hydrolysing a 50/50 polyester/cotton fabric, the effective solid loading is lower compared to hydrolysis of pure cotton fabric, leading to a higher glucose yield. Similar results were found by Leenders *et al.* showing that increasing the solid loading decreased the glucose yield.^57^ This phenomenon is known as the high-solid effect and is caused by a number of factors, ranging from product and inhibitor (mainly in enzymatic hydrolysis) concentrations, mixing efficiency, water availability and mass transfer limitations.^76^

In general, a pretreatment seems to be less important for acid hydrolysis compared to enzymatic hydrolysis, where it is essential.^15^ From the 11 considered studies, only three utilised a pretreatment. With a sufficient acid concentration, the acid penetrates the recalcitrant structure of the cellulose fibres, allowing for the cellulose to hydrolyse into glucose.

#### 3.1.5 Solid loading

The solid loading of all the mentioned experiments is ranging between 30 and 50 g L^−1^, with a cotton loading ranging from 0.2 to 50 g L^−1^. Typical, large scale processes operate at a solid loading of 0.1–0.3 kg L^−1^, to increase the sustainability and economics of the process. However, experimental data also shows that increasing the solid loading decreases the monomeric glucose yield, which is in line with the high-solid effect earlier mentioned in this paper. Therefore, all studies would need additional research to find the optimal reaction conditions at higher solid loadings.

#### 3.1.6 Reaction time

The reaction time of the considered studies ranged from 50 min to 24 h, with most studies performing the experiment within 2 h. Kawamura *et al.* performed the dilute acid reaction with 1 wt% citric acid for 50 min, leading to a glucose yield of 22% and 15% HMF, which was the actual focus of their research.^66^ The reaction time of the Leenders study was substantially longer compared to other studies (18 h).^57^ However, the absence of any additional heating distinguishes this work as a glucose yield of 80% was obtained with 43 wt% HCl already at room temperature.

#### 3.1.7 Process sustainability

Recycling polycotton waste has an enormous beneficial impact on reducing the environmental burden currently caused by the textile industry. The valorisation of polycotton waste also aligns with Sustainable Development Goal (SDG) 12.5; ‘By 2030, substantially reduce waste generation through prevention, reduction, recycling and reuse’.^77^ However, the environmental impact of the technologies used to valorise polycotton waste must also be assessed, as SDG 9 strives to ‘Build resilient infrastructure, promote inclusive and sustainable industrialisation and foster innovation’.^78^

One way to evaluate the sustainability of a process is by assessing the energy consumption during the reaction. To provide an initial indication of energy consumption during valorisation, only the energy consumption during pretreatment and hydrolysis was considered. Energy consumption related to transport and size reduction was assumed to be similar in all the studies. Although energy consumption during downstream processing will differ per study, this was not included due to the lack of available data. Thus, the energy consumption to heat and sustain the reaction temperature during the pretreatment and hydrolysis for a 1 litre scale was calculated for all studies. Further details on the calculation and assumptions made can be found in the ESI, Section 3.†

Table 1 also presents the energy consumption per study, which ranges from 0 to 233 Wh. The study by Leenders *et al.* required no energy input as the concentrated acid hydrolysis was performed at room temperature (Table 1, entry 9). During the concentrated acid hydrolysis performed by Chu *et al.* and Sun *et al.* (Table 1, entries 3 and 10), a relative low energy consumption was observed (12 Wh and 30 Wh, respectively) as they perform the hydrolysis at lower reaction temperature (40 and 65 °C) and no pretreatment was needed.

For the studies performing a concentrated acid pretreatment followed with a dilute acid hydrolysis (Table 1, entries 1, 2 and 4), a relatively higher energy consumption was observed as the diluted acid hydrolysis was performed at elevated temperatures (116, 91 and 192 Wh). As the cellulose is solubilised during the concentrated acid pretreatment, the subsequent conversion of cotton to glucose in the dilute acid hydrolysis generally requires less energy. Without this solubilisation step, more energy is needed during the dilute acid hydrolysis, resulting in a high energy consumption. Therefore, the dilute acid hydrolysis studies (without pretreatment), in general, are the most energy-intensive. The study by Kawamura *et al.* (Table 1, entry 11), which performed dilute acid hydrolysis at 225 °C recorded the highest energy consumption, namely 233 Wh.

Thus, processes using concentrated acid hydrolysis utilise the lowest energy consumption and are the most sustainable in this regard. Notably, the study by Leenders *et al.* (Table 1, entry 9) also obtained relatively high glucose yields (80%), suggesting that this method may be a sustainable approach to textile waste valorisation. Additionally, energy consumption increases when a concentrated acid pretreatment is followed by a diluted acid hydrolysis and the highest energy is observed when performing a diluted acid hydrolysis.

However, the use of energy during pretreatment and hydrolysis is merely one indicator on the sustainability of a process. As mentioned, the energy required for downstream processing was not included due to the lack of data for a reliable comparison.

The use of green metrics, such as process mass intensity (PMI), is also valuable, as it compares the total mass of the materials used in the process relative to the mass of the product. Unfortunately, insufficient data was available to calculate the PMI for all the studies.

#### 3.1.8 Limitations

Lastly, definitive conclusions can not be made when comparing these methods, due to the variability in starting materials. Although all materials contain cellulose, due to different harvesting, spinning and downstream processes the cellulose structure can substantially be altered leading to a difference in reactivity of the cellulose chains. McAlister and Rogers found that spindle-picked cotton had a higher fibre strength, fibre length and uniformity index compared to stripper-harvested cotton.^79^ Furthermore, Mathangadeera *et al.* found that the mechanical stress performed on the cotton fibre during spinning led to the dislocation of microfibrils, making them more accessible during chemical reactions.^80^ Additionally, the use of dyes and additives could potentially inhibit the reaction and the effect of wear and tear during the lifetime of the fabric alters its properties. Palme *et al.* found that extensive laundering (>50×) of cotton sheets decreased its specific surface area by 40% caused by an increase in coalescence of cellulose fibrils, which could have a negative effect on the accessibility and reactivity of cellulose.^81^ Therefore, definitive conclusions cannot be made and additional research to the properties of the cotton from different stages from its production process and lifetime could provide more insights into the reactivity of the different materials.

### 3.2 Enzymatic hydrolysis

**Fig. 6:**
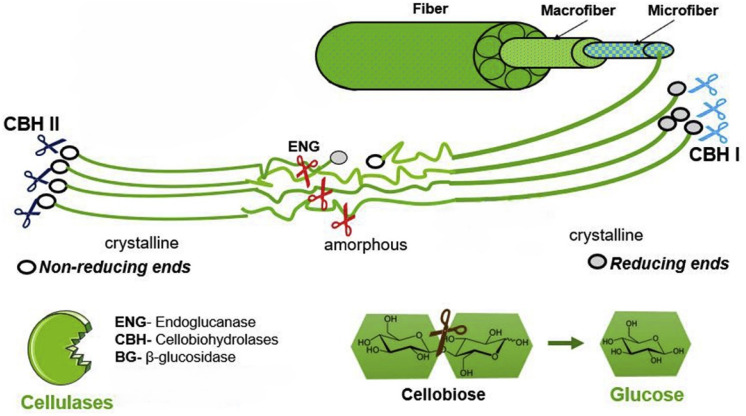
Synergistic working of cellulases. Endoglucanases (ENG) randomly cleave the cellulose chains. Cellobiohydrolases (CBH) cleave the chain ends. β-Glucosidases (BG) cleave oligomers (≤6 AGU) into glucose. Reproduced from Houfani *et al.* (2020).^82^ Copyright (2020) with permission from Elsevier.

The enzymatic hydrolysis of cotton is performed by cellulase enzymes. Cellulases are *O*-glycoside hydrolases that hydrolyse the β-1,4-glycan bond of cellulose resulting in glucose, cellobiose (glucose dimer) and cellooligosaccharides.^82,83^ Cellulases are a combination of multiple enzymes; endo- and exoglucanases, and β-glucosidases, that work synergistically and simultaneously (Fig. 6).^82^ Endoglucanases randomly break amorphous cellulose chains *via* cleavage of β-1,4-glycosidic bonds.^84^ However, some endoglucanases can break down the crystalline cellulose chains.^85,86^ Exoglucanases or cellobiohydrolases (CBH) break down the cellulose chains into cellobiose, where CBHI and CBHII attack the reducing and nonreducing ends, respectively.^85,87^ β-Glucosidases hydrolyse cellobiose and in some cases cellooligosaccharides with up to 6 repeating AGU units into single glucose molecules.^82,88^

The enzymatic hydrolysis mechanism consists of three stages: the adsorption of cellulase on the cellulose surface, the biodegradation of cellulose into sugars and the desorption of cellulase.^15^ The rate determining step is enzyme absorption, which is a function of the enzyme concentration, the available surface area of cellulose, the physical properties of the enzyme and the hydrolysis conditions.^15^ Cellulases can be combined with fermenting enzymes in a single reactor vessel for the simultaneous saccharification and fermentation process or in separate vessels for the saccharification and separate fermentation process.^82^ In these processes, glucose is used to produce bioethanol, biohydrogen and lactic acid *via* fermentation, but glucose can also be used for the production of biogas *via* anaerobic digestion.^82^

In Table 2, the state of the art methods are presented for the enzymatic hydrolysis of (poly)cotton materials to glucose without polyester destruction.

**Table 2:** Enzymatic hydrolysis methods for the depolymerisation of cotton to glucose without destruct polyester

Entry	Enzymes	PET/cotton ratio	Textile	Pretreatment	Enzyme loading	Solid (cotton) loading [g L^−1^]	Temp. [°C]	Reaction time [h]	Molar glucose yield [%]	Suitable for polycotton valorisation	Energy consumption [Wh]	References
1	Cellulases	0/100	White cotton shirt	Mechanical refining: 10 800 revs in PFI mill	6 FPU per g	50	50	96	97.1 conv	No	135	Vera *et al.*, 2023a (ref. 120)
2	Cellulases	0/100	Black cotton shirt	Mechanical refining (1) followed by ozone aided alkaline pretreatment (2): (1) 10 000 revs in PFI mill. (2) O_3_ (3.2 wt%), NaOH (2 w/v%) + H_2_O_2_ (1 w/v%), solid loading 3 wt%, 70 °C, 60 min	6 FPU per g	50	50	96	90	No	188	Vera *et al.*, 2023b (ref. 119)
3	Cellulases	0/100	White cotton	Alkaline pretreatment: NaOH (15 w/v%), solid loading 0.01 g mL^−1^, RT, 0.5 h	20 FPU per g	10	45	120	89.1^a^	Yes	156	Cho *et al.*, 2023 (ref. 17)
4	Cellulases	0/100	Purple bed sheets	Dissolution: [AMIM]Cl, solid loading 0.1 g g^−1^, 110 °C, 500 rpm, 75 min	8.1 IU per mL	40	50	48	85^a^	No	182	Guo *et al.*, 2016 (ref. 115)
5	Cellulases	0/100	Undyed cotton shirt	Dissolution: [AMIM]Cl, solid loading 0.01 g g^−1^, 110 °C, 500 rpm, 90 min	66 IU per g	50	50	24	73.5^a^	No	156	Hong *et al.*, 2012 (ref. 114)
6	Cellulases	0/100	Bleached cotton	Corona pretreatment: atmospheric pressure, 6 h, 900 W	N.A.	32	50	168	33.7	No	5614	Nikolić *et al.*, 2016 (ref. 98)
7	Cellulases	0/100	Mercerised cotton	—	N.A.	33	50	192	32.2	Yes	240	Nikolić *et al.*, 2017 (ref. 113)
8	Cellulases	0/100	Bleached cotton	Ultrasonic pretreatment: 20 kHz, 60% amplitude, 32.2 W cm^−2^, 90 min, 500 W	44 FPU per g	7.5	50	1.5	7.4^a^	Yes	781	Szabo and Csiszar, 2017 (ref. 137)
9	Cellulases	20/80	Textile with reactive dye	Selective alkaline dissolution: NaOH (7 w/v%) + urea (12 w/v%), −20 °C, 6 h	25 FPU per g	1.6 (1.28)	50	96	77.2	Yes	193	Hu *et al.*, 2018a (ref. 87)
10	Cellulases	20/80	Textile	Selective alkaline dissolution: NaOH (7 w/v%) + urea (12 w/v%), −20 °C, 6 h	1.56 FPU per g	N.A. (N.A.)	N.A.	96	70.2	Yes	193	Hu *et al.*, 2018b (ref. 100)
11	Cellulases	40/60	Blue shirt	Acid pretreatment: H_3_PO_4_ (85 wt%), solid loading 5.2 wt%, 50 °C, 100 rpm, 2 h	10 FPU per g	20 (12)	50	48	99^a^	Yes	97	Kuo *et al.*, 2014 (ref. 109)
12	Cellulases	40/60	Blue shirt	Acid pretreatment: H_3_PO_4_ (85 wt%), solid loading 5 wt%, 50 °C, 1 h	10 FPU per g	20 (12)	50	12	93^a^	Yes	56	Kuo *et al.*, 2010 (ref. 99)
13	Cellulases	50/50	Bed linen	Steam explosion (1) followed by alkaline pretreatment (2): (1) 150 °C, 5 bar, 10 min. (2) 4 w/v% NaOH, 4 °C, overnight	N.A.	N.A. (N.A.)	50	168	68	Yes	397	Gritsch *et al.*, 2023 (ref. 106)
14	Cellulases	60/40	Textile with reactive dye	Selective alkaline dissolution: NaOH (12 w/v%) + urea (7 w/v%), −20 °C, 6 h	25 FPU per g	N.A. (N.A.)	50	96	44.6	Yes	193	Wang *et al.*, 2018 (ref. 101)
15	Cellulases, β-glucosidases	N.D./93	Blue jeans	Acid pretreatment: H_3_PO_4_ (>82 wt%), solid loading 0.125 g mL^−1^, 50 °C, 90 rpm, 0.5–1 h	20 FPU per g, 30 IU per g	30 (27.9)	45	48	60.7	Yes	90	Jeihanipour and Taherzadeh, 2009 (ref. 97)
16	Cellulases, β-glucosidases	N.D./88	Towels	MW assisted acid pretreatment: H_2_SO_4_ (0.5 wt%), solid loading 0.025 g mL^−1^, 200 °C, 2.45 GHz, 7 min	45 FPU per g, 53 IU per g	20 (17.6)	50	72	80.7	No	312	Sasaki *et al.*, 2019 (ref. 110)
17	Cellulases, β-glucosidases	N.D./88	Towels	Acid pretreatment (1) followed by MW assisted hydrolysis (2): (1) H_2_SO_4_ (51 wt%), 0.025 g mL^−1^, RT, 30 min. (2) Residue from acid pretreatment in 20 mL H_2_O, 180 °C, 2.45 GHz, 3 min	22 FPU per g, 26 IU per g	20 (17.6)	50	72	76	No	288	Sasaki *et al.*, 2020 (ref. 111)
18	Cellulases, β-glucosidases	14/86	Jeans	Acid pretreatment: H_3_PO_4_ (85 wt%), solid acid ratio 1 : 15, 50 °C, 130 rpm, 7 h	7.5 FPU per g, 15 CBU per g	10 (8.6)	50	96	91	Yes	155	Shen *et al.*, 2013 (ref. 12)
19	Cellulases, β-glucosidases	14/86	Jeans	Acid pretreatment: H_3_PO_4_ (85 wt%), solid loading 6.25 wt%, 50 °C, 7 h	15 FPU per g, 30 IU per g	10 (8.6)	50	96	83^a^	Yes	155	Zhujun *et al.*, 2015 (ref. 138)
20	Cellulases, β-glucosidases	40/60	White textile	Selective alkaline dissolution: NaOH (7 w/v%) + urea (12 w/v%), solid loading 5 wt%, −20 °C, 1 h	30 FPU per g, 60 IU per g	30 (18)	45	72	91	Yes	156	Gholamzad *et al.*, 2014 (ref. 102)
21	Cellulases, β-glucosidases	40/60	Textile with blue reactive dye	Selective alkaline dissolution: NaOH (7 w/v%) + urea (12 w/v%), solid loading 5 wt%, −20 °C, 6 h	20FPU per g, 10 IU per g	30 (18)	50	96	98.3	Yes	193	Li *et al.*, 2019 (ref. 95)
22	Cellulases, β-glucosidases	50/50	Orange textile	Selective dissolution: NMMO (85 wt%), solid loading 0.05 g mL^−1^, 120 °C, 2 h (washed 2nd time at 120 °C when cellulose remaining in textile)	20 FPU per g, 30 IU per g	N.A. (N.A.)	N.A.	48	91	Yes	194	Jeihanipour *et al.*, 2010 (ref. 117)

a Reducing sugar yield.

#### 3.2.1 Enzyme loading

The catalytic activity of general enzymes is expressed in international unit (IU). The International Union of Pure and Applied Chemistry defines the IU as “the amount of enzyme that catalyses the conversion of one micromole of substrate per minute under the specified conditions of the assay method”.^89^ The IU is determined by a standard assay where the substrate yield is measured under standardised reaction conditions of time, temperature and pH. For cellulase, a specific assay was designed to measure the cellulase activity in terms of filter paper units (FPU).^90^ A FPU is defined as the quantity of enzymes that releases one micromole of glucose per minute per millilitre from a piece of Whatman's No. 1 filter paper as substrate under the defined assay conditions.^90,91^ When the enzyme has a high catalytic activity, the FPU will be small and *vice versa*. Thus, depending on the assay method, the enzyme loading of cellulase is either given in FPU per g cellulose or IU per g cellulose. Additionally, β-glucosidase activity can be determined using a standard protocol with a cellobiose substrate and resulting activities are expressed in cellobiase units (CBU).^91,92^ A CBU is defined as the enzyme amount which converts one micromole of cellobiose to two micromoles of glucose per minute per millilitre. The β-glucosidase can be expressed in CBU per g cellulose or IU per g cellulose.

The cellulases loading used ranged from 1.56 FPU per g cellulose to 66 IU per g cellulose where twelve studies used a concentration above 20 FPU per g cellulose, which is considered a high enzyme concentration.^15^ Such high enzyme loadings makes enzyme recycling essential when the process would be performed on a large scale, due to the high cost of enzymes.

Additionally, a substantial number of the studies performed the enzymatic hydrolysis with an additional amount of β-glucosidases. Cellobiose is known for its strong inhibiting effect on the activity of cellulases, therefore, additional β-glucosidases are added to decrease the concentration of cellobiose.^93–96^ Half of the studies that used cellulases with additional β-glucosidases (four out of eight) obtained a glucose yield of 91% or higher. Although the addition of extra β-glucosidases will positively affect the yield of the process and thus improve the economical aspect of such a process, the cost of the additional enzymes will also needed to be taken into account when considering the overall process economics.

#### 3.2.2 Textile usage

The studies considered used a variety of textiles for the enzymatic hydrolysis, ranging from 0/100 to 60/40 polyester/cotton. Eleven studies focused on the processing of polycotton materials with varying polyester cotton ratios, in contrast to the acid hydrolysis studies mentioned in Table 1. Extensive lab research on the enzymatic hydrolysis of cotton from polycotton materials has been performed. With the right pretreatment, the enzymatic hydrolysis yields are in general higher compared to acid hydrolysis, making it an interesting feature for a polycotton waste treatment process.^10,99^

#### 3.2.3 Pretreatment

Enzymatic hydrolysis of cotton leads to a low glucose yield when no pretreatment is performed. The primary objective of a pretreatment is to reduce the crystallinity of the cellulose and increase the surface area and enzyme accessibility.^17,97,98^ When hydrolysing polycotton materials, separation of polyester and cotton fraction is also an important objective. The pretreatments in this review range from chemical pretreatments, including the use of metal alkalines (*e.g.* NaOH) combined with urea, or acids (*e.g.* H_2_SO_4_, H_3_PO_4_) or solvents (*e.g.* ionic liquids 1-allyl-3-methylimidazolium chloride ([AMIM]Cl) and *N*-methylmorpholine *N*-oxide (NMMO)) to dissolve and precipitate cellulose,^15^ to mechanical pretreatments, such as the use of ultrasound mechanical refining. The reduced degree of crystallinity and a high specific surface area after pretreatment improve the enzymatic hydrolysis performance.^99^ The pretreatment does not remove the dyes, however, it does increase the accessibility of the cellulose to cellulases.^99^

##### 3.2.3.1 Alkaline based pretreatments

Considering the pretreatments in Table 2, chemical pretreatment for (poly)cotton materials is the most used method, more specific the concentrated alkaline method with a NaOH/urea solution at −20 °C for 1 or 6 h (Table 2, entries 9, 10, 14, 20 and 21).^87,95,100–102^ Sodium hydroxide, also used for the mercerisation of cotton, is an intracrystalline swelling agent that disrupts the intramolecular hydrogen bonds, causing a conformational change of the cellulose in cotton.^103^ This leads to a crystallinity change of cellulose from cellulose I to II. Urea stabilises and solubilises cellulose in aqueous solution by preventing the aggregation of cellulose as urea accumulates near the hydrophobic regions.^104^ Thus, urea has no direct interaction with cellulose, it merely stabilises the swollen cellulose structures so NaOH can penetrate into the crystalline regions.^105^ After dissolution and separation from the polyester fibres, the cellulose fibres were subjected to enzymatic hydrolysis with varying enzyme loadings. Hu *et al.* performed the enzymatic hydrolysis with low enzyme loading of 1.56 FPU per g cellulases which led to a glucose yield of 70.2% (Table 2, entry 10).^100^ Li *et al.* obtained the highest glucose yield of 98.3% by combining the alkaline pretreatment with enzymatic hydrolysis (20 FPU per g cellulases + 10 IU per g β-glucosidases) (Table 2, entry 21).^95^ Although this work led to the highest glucose yield, the high enzyme loading in combination with the two-step recycling process will negatively impact the economic viability of this process. All other studies that used alkaline pretreatments for (poly)cotton materials also performed the enzymatic hydrolysis with very high enzyme loading (25 FPU per g cellulases or more).^87,101,102^ Cho *et al.* studied the effect of solely NaOH solution (15 w/v%) at room temperature for 0.5 h, combined with enzymatic hydrolysis, and obtained a glucose yield of 89.1% (Table 2, entry 3).^17^ Gritsch *et al.* combined alkaline treatment with steam explosion followed by enzymatic hydrolysis which resulted in a glucose yield of 68% (Table 2, entry 13).^106^ Steam explosion defibrillates cellulose by the rapid expansion of steam and increases the accessibility for the enzymes.^106^ The pretreatment of cotton with steam explosion is usually performed at temperatures of 190–225 °C, however, as these higher temperatures would destruct the polyester fibres pretreatment was performed at 150 °C.^106^ By performing the steam explosion at 150 °C the PET remains intact, yet, the pretreatment is less effective in treating cotton, leading to a lower glucose yield after enzymatic hydrolysis.^106^

Additionally, although it is known that polyester hydrolyses in alkaline conditions, several researches found that when the right conditions were chosen the PET hydrolysis is minimised. Gritsch *et al.* found that pretreatment with a 4 w/v% sodium hydroxide concentration did not lead to a significant mass loss when treating PET fibres and detected 0.17 mM of terephthalic acid with high-performance liquid chromatography.^106^ Gholamzad *et al.* found that during the pretreatment of polycotton fabrics with 12 w/v% NaOH at −20 °C for 1 h led to a small decrease in crystallinity and molecular weight of the PET fibres.^102^ They stated that the NaOH only affects the PET fibres *via* topochemical reactions and that the treatment is not able to affect the core of the PET fibres.^107^ Similar results were obtained by Li *et al.* and Hu *et al.*^95,100^ Additionally for alkaline hydrolysis of PET, a temperature of at least 80 °C or higher are commonly used to obtain sufficient yields.^108^ Thus, operating at these lower temperatures, alkaline pretreatments can be used when valorising polycotton waste textiles.

##### 3.2.3.2 Acidic pretreatments

Acidic pretreatments are also commonly used on (poly)cotton materials. The different reactivities of cotton and polyester lead to a convenient separation of both fractions. Concentrated acid pretreatment is used to dissolve the crystalline cellulose. All studies that consider solely acidic pretreatment in Table 2 use phosphoric acid (Table 2, entries 11, 12, 15, 18 and 19). By pretreating cellulose with phosphoric acid, the crystalline cellulose is disrupted and the resultant amorphous cellulose formed makes it possible to completely enzymatically hydrolyse the cotton.^97^ The acid pretreatment is performed with 85 wt% H_3_PO_4_ (14.7 M) at 50 °C for various reaction times ranging from 1 to 7 h, with yields ranging from 60.7 to 99%.^12,97,99,109^ Besides these high yields, Shen *et al.* found that pretreatment of polycotton material with 85 wt% H_3_PO_4_ at 50 °C for 7 h led to full polyester recovering,^12^ making pretreatment with a high concentration H_3_PO_4_ suitable for cellulose solubilisation without polyester destruction.

Sasaki *et al.* performed two studies combining acidic pretreatment with microwave treatment (Table 2, entries 16 and 17).^110,111^ Both studies use sulfuric acid, one with a low H_2_SO_4_ concentration at elevated temperature,^110^ and one with concentrated H_2_SO_4_ at room temperature.^111^ For the enzymatic hydrolysis, enzyme loadings of 45 FPU per g cellulases + 53 IU per g β-glucosidases or 22 FPU per g cellulases + 26 IU per g β-glucosidases led, in 72 h, to a glucose yield of 80.7 and 76%, respectively.^110,111^ Often pretreatments using harsh temperature and pressure conditions are not preferred as these can lead to the production of HMF, which can inhibit the enzymatic hydrolysis.^82^ Additionally, it is not expected that the pretreatment is suitable for polycotton materials, due to the use of sulfuric acid and PET susceptibility to H_2_SO_4_.

The reaction time of alkaline pretreatment is often much longer compared to acidic pretreatment as the reaction temperature is substantially lower. At lower temperatures, the ionic hydrates (Na^+^ and OH^−^) decrease their mobility and increase their residence time around the cellulose chain, resulting in a higher penetration rate of the solvent.^99,102,103,112^

##### 3.2.3.3 Selective dissolution

The selective dissolution method is another chemical method used to decrease the crystallinity of cellulose.^17,113^ Imidazolium-based chloride ionic liquids (IL) are known for their cellulose dissolving properties with [AMIM]Cl being less toxic and more effective compared to 1-butyl-3-methylimidazolium chloride ([BMIM]Cl).^114^ Guo *et al.* found that pretreatment with [AMIM]Cl significantly reduced the crystallinity and disrupted the bonds between dye molecules and cellulose.^115^ The ionic liquid treatment was combined with enzymatic hydrolysis with moderate enzyme loading (8.1 IU per mL cellulases) which resulted in a reducing sugar yield of 85% compared to a reducing sugar yield of 27% without pretreatment (Table 2, entry 4).^115^ Hong *et al.* used a similar pretreatment but performed the enzymatic hydrolysis at a much higher enzyme loading (66 IU per g cellulases) which led to a reducing sugar yield of 73.5% (Table 2, entry 5).^114^

Yet, the effect of [AMIM]Cl on PET is unclear. Sun *et al.* investigated the dissolution of cellulose from polycotton materials with ionic liquids.^116^ Although they did not investigate [AMIM]Cl, they did concluded that 1-butyl-3-methylimidazolium chloride and 1,3-dimethylimidazolium dimethyl phosphate at 130 °C for 3–10 hours were both able to dissolve cotton while preserving PET. Thus, further research to the behaviour of PET in [AMIM]Cl would be needed to be conclusive, however, the prospect is positive.

Moreover, Jeihanipour *et al.* studied the effect of selective dissolution with NMMO followed by enzymatic hydrolysis (20 FPU per g cellulases + 30 IU per g β-glucosidases) resulting in a 91% glucose yield (Table 2, entry 22).^117^ Therefore, NMMO seems more effective as pretreatment compared to [AMIM]Cl, which could be caused by the increased swelling caused by NMMO compared to [AMIM]Cl.^118^ Additionally, Jeihanipour *et al.* mention that the polyester is not affected by the pretreatment with NMMO, making it a suitable method for polycotton valorisation.^117^

Although ionic liquids have a broad liquid region, high thermal stability and negligible vapor pressure, their high viscosity and potential deactivation of enzymes makes it inapplicable on large scale.^12^ Additionally, the reuse of the IL is very challenging, as a large amount of antisolvent (such as water) is needed to recover cellulose from the IL. As an IL is highly soluble in water, recovery of the IL is very costly making this process unviable on large scale.

Thus, while high yields can be obtained when enzymatic hydrolysis is combined with chemical pretreatment, the high quantity of chemicals needed during the pretreatment and large amounts of enzymes challenges the overall process on the economical and environmental impact.

##### 3.2.3.4 Mechanical pretreatment

Mechanical pretreatment increases surface area by reducing the particle size. The surface area of cellulose is the key factor in increasing the yield for enzymatic hydrolysis.^15^ In both studies of Vera *et al.*, a mechanical refining was used to aid the enzymatic hydrolysis. In one study, mechanical refining was combined with an ozone aided alkaline pretreatment to hydrolyse a black cotton t-shirt, where the ozone aided alkaline pretreatment was able to fully remove the black dye (Table 2, entry 2). This method resulted in a 90% cotton conversion.^119^ When only mechanical refining was combined with enzymatic hydrolysis of a white cotton shirt, a cotton conversion of 97.1% was obtained (Table 2, entry 1).^120^ However, this method would be less convenient for polycotton samples as the mechanical refining would lead to small PET fibres.

Szabo and Csisbar used an ultrasonic treatment coupled with enzymatic hydrolysis (44 FPU per g cellulases) resulting in the lowest obtained glucose yield of 7.4% (Table 2, entry 8). However, the reaction time of the enzymatic hydrolysis was only 1.5 h, which is the shortest of all studies. Therefore, to establish whether the low glucose yield is a result of the ineffective pretreatment or of the short reaction time, further research is needed.

Gotoh and Harayama used ultrasound (38 kHz) to clean PET fabrics and reported little damage to the fabric.^121^ Thus, initially ultrasonic pretreatment seems to be applicable for polycotton materials, however, it should be tested whether the PET also remains undamaged after the higher frequency.

Nikolić *et al.* did not perform a pretreatment at all in their 2017 study. A direct enzymatic hydrolysis of fully mercerised cotton (cellulose II) for 8 days led to a glucose yield of 32% (Table 2, entry 7).^113^ It is known that mercerisation leads to a lower crystallinity due to the transformation of cellulose I to II and an increased surface area.^112,113,122^

The corona treatment on the PET surface chemically and physically modifies the surface resulting in the formation of cavities and bumps.^123^ However, it is unknown how this will affect other properties of PET thus more research is needed to confirm the suitable fit for polycotton valorisation.

To conclude, the studies using (steam explosion aided) alkaline dissolution pretreatment (Table 2, entries 3, 9, 10, 13, 14, 20 and 21), ultrasonic pretreatment (Table 2, entry 8), acid pretreatment with H_3_PO_4_ (Table 2, entries 11, 12, 15, 18 and 19), selective dissolution pretreatment with NMMO (Table 2, entry 22) or no pretreatment (Table 2, entry 7) are suitable for polycotton waste valorisation as they only selectively dissolve cotton without PET destruction.

#### 3.2.4 Solid loading

All studies that enzymatically hydrolysed (poly)cotton materials (Table 2) were conducted with low solid loadings. The solid loadings ranged from 1.6–50 g L^−1^ (0.16–5 wt%), with the cotton fraction ranging from 1.28 to 50 g L^−1^. Li *et al.* studied the effect of substrate loading with 40/60 polyester/cotton textile and found that a substrate loading higher than 3 w/v% had a negative effect on the glucose yield.^95^ The produced glucose had an inhibitory effect on the hydrolytic reaction as it inhibited the binding of the cellulase.^93,94^ However, to which extent and with which mechanism the adsorption is inhibited is still unknown. Furthermore, a high monosaccharide concentration reduces the presence of free water molecules which could inhibit the cellulases.^95,124^

#### 3.2.5 Process conditions

All studies considered performed the enzymatic hydrolysis at a reaction temperature of 45 or 50 °C. Above 60 °C, most enzymes denature and the hydrolysis yield decreases.^95^ The reaction time was not as uniform as the reaction temperature varied from 1.5 to 192 h, with the highest yield (98.3%) obtained after 96 h by Li *et al.* (Table 2, entry 21).^95^ When considering the reaction time with regard to the glucose yield, no obvious trend is visible.

#### 3.2.6 Process sustainability

As mentioned in Section 3.1.7, the environmental impact of the technology used to valorise the polycotton waste should also be assessed. Table 2 presents the energy consumption for all enzymatic hydrolysis studies, which ranges from 56 to 5614 Wh. Half of the studies report an energy consumption between 100 and 200 Wh (13 out of the 22 considered studies), while six studies having an energy consumption above 200 Wh. The work of Nikolić *et al.* (Table 2, entry 6) recorded the highest energy consumption at 5614 Wh, due to the use of a highly energy-intensive corona pretreatment for 6 h. Only three studies utilise an energy consumption below 100 Wh (Table 2, entries 11, 12 and 15), which all use an H_3_PO_4_ acid pretreatment.

Because enzymatic hydrolysis typically requires an energy-intensive pretreatment and a longer reaction time compared to acid hydrolysis, its overall energy consumption is on average higher than the energy consumption of acid hydrolysis processes. Nevertheless, enzymatic hydrolysis generally obtains a significantly higher yield compared to acid hydrolysis. When considering both the energy usage and the yield, the studies by Kuo *et al.* (Table 2, entries 11 and 12) obtain the highest (reducing sugar) yield of 99% and 93% with the lowest energy consumption (97 and 56 Wh), respectively.

In summary, most enzymatic hydrolysis studies have an energy consumption between 100 and 200 Wh. The studies by Kuo *et al.* (Table 2, entries 11 and 12) are the most energy effective by obtaining high yields with the lowest energy consumption.

#### 3.2.7 Limitations

The primary limitation of enzymatically hydrolysing polycotton textiles is that the dyes present in the fabric negatively impact the enzyme performance. It has been reported that the dyes inhibiting the enzymes due to the dyes interaction with the cellulose surface.^13,15,99,125^

The extent of the inhibition depends on the chemical structure of the dye and the dye concentration.^125–128^ Buschle-Diller and Traore investigated the effect of the molecular size of direct dyes on the performance of enzymatic hydrolysis of cotton.^128^ They considered four direct dyes of varying molecular sizes: C.I. Direct Red 81 (DR81), C.I. Direct Red 16, C.I. Direct Blue 1, and C.I. Direct Green 26 (DG26), with DR81 having the smallest molecular size and DG26 the largest. They found that all samples treated with the direct dye for 24 hours had a lower hydrolysis yield compared to the undyed sample. However, no direct correlation was found between the size of the dye molecule and the inhibition of enzymatic activity.

Buschle-Diller and Traore also examined the performance of five reactive dyes, including three monofunctional reactive dyes—C.I. Reactive Yellow 3 (RY3), C.I. Reactive Yellow 17 (RY13), and C.I. Reactive Blue 19 (RB19)—and two bifunctional reactive dyes—C.I. Reactive Red 120 (RR120) and C.I. Reactive Black 5 (RB5).^128^ Reactive dyes form a covalent bond which may create a barrier for the enzymes.^122,125,126^ Since commercial reactive dyes for fibres typically contain two major reactive groups—triazine (RY3, RR120) and vinyl sulfone (RY13, RB19, RB5)—dyes from both groups were included in the study. For the monofunctional reactive dyes, all three dyes led to a decrease in hydrolysis yield compared to the undyed sample. However, there was no clear indication that the type of reactive group (triazine or vinyl sulfone) played a significant role.

When assessing the hydrolysis yield of the two bifunctional reactive dye-treated samples, they found that both dyes inhibited the hydrolysis reaction. However, the inhibition caused by RB5 was more severe compared to RR120, which results were comparable to those of the monofunctional reactive dyes. Comparing the chemical structure of RB5 and RR120 shows an important difference in location of the reactive groups. The reactive groups of RR120 are located at the centre of the molecule, where the reactive groups of RB5 are located at the outer ends of the molecule. Buschle-Diller and Traore assumed that RR120 forms only one reactive bond with the hydroxyl group, thus acting as a monofunctional reactive dye. In contrast, they concluded that RB5 can form crosslinks within the cellulose structure by reacting with both groups, thereby shielding the cellulose from enzymatic attack.

Cavaco-Paulo and Almeida also found that inhibition of the enzymatic hydrolysis occurs with reactive dyes.^125^ They tested five different reactive dyes (C.I. Reactive yellow 26, C.I. Reactive yellow 160, C.I. Reactive Blue 109, Procion Yellow HE-XL and Marine Cibacrone FG) and found inhibition for all the samples after 1 hour. They also considered a sulphur (C.I. Solubilised Sulphur Red 11) and vat (C.I. Vat Red 10) dye, which had a minor effect on the weight loss caused by enzymatic hydrolysis.^125^

Koo *et al.* stated that the vat dye is not large enough to hinder the cellulases or form a dye–enzyme complex.^127^ This is questionable as the size of a vat dye molecule used (C.I. Vat Blue 1) is in the same order as glucose, which could result in inhibiting the binding of the substrate to the catalytic site. A possibility could be that vat dyes do not solubilise during the reaction, as vat dyes are insoluble in water, whereas the cellulose is solubilised during the pretreatment so it can be enzymatically hydrolysed. However, more in depth research to the behaviour of vat dyes under the reaction conditions used during enzymatic hydrolysis would be needed to draw any conclusions. Koo *et al.* found similar results as Buschle-Diller and Traore, and Cavaco-Paulo and Almeida on the inhibiting effect of direct and reactive dyes on the enzymatic hydrolysis.^127^ The results of Guo *et al.* also showed a decrease in cellulases activity when using reactive dyes, however, the effect was less profound.^115^

Concluding, the scientific community has shown that reactive and direct dyes hinder cellulases during the hydrolysis of cotton.

The effect of sulphur and vat dyes on cellulases is less inhibiting, however, more research would be needed to better understand the effect of the sulphur and vat dyes on the cellulases activity.

## 4 Large scale recycling

To tackle the enormous amounts of blended waste textile, it is important that any recycling method is not only effective on laboratory but also on large scale. For a process to be viable on a large scale, aspects such as its technical feasibility, sustainability, economic viability as well as safety aspects need to be considered.

### 4.1 Technical feasibility

The scale up from lab experiment to pilot plant requires extensive research to ensure that the process is efficient on a larger scale as well. Loo *et al.* discussed the technology readiness level (TRL) of acid hydrolysis and enzymatic hydrolysis, which indicates the maturity level of a technology. It was stated that the TRL of acid and enzymatic hydrolysis of blends is 5–6 and 5, respectively, indicating that both technologies have been tested in relevant environments.^4^ Leenders *et al.* investigated the concentrated hydrochloric acid hydrolysis of actual post-consumer polycotton waste textile in a 230 L reactor in Avantium's Dawn Technology pilot plant, confirming the TRL of 6.^57^ Besides Avantium's Dawn Technology, there is no large scale process that is able to convert cotton (from polycotton) waste with high selectivity into glucose *via* acid hydrolysis. Blocktexx does perform dilute acid hydrolysis of polycotton waste on a 10 kt scale, however, the goal is to obtain CellTexx, a cellulose clay containing microcrystalline cellulose, instead of glucose.^129^ Enzymatic hydrolysis of textile has been done on larger scale, for instance by a cooperation between Cotton Incorporated and North Carolina State University. Textile was pretreated with dilute phosphoric acid at elevated temperatures whereafter the cellulose was hydrolysed with a combination of cellulases and β-glycosidases. The process is executed with 23 kg per run,^130^ indicating that the TRL of enzymatic hydrolysis for (poly)cotton waste is also 6. Large scale enzymatic processes for other fibres are also existing. Carbios is able to enzymatically hydrolyse 100% white polyester textiles after a pretreatment that reduces the crystallinity from the polyester.^131^ The effect of the enzymatic hydrolysis on coloured textiles has not been mentioned. Once fully operational, Carbios PET biorecycling plant in Longlaville, France, would be able to enzymatically recycle 50 kt of PET on a yearly basis.^132^ However, in December 2024 Carbios announced that the construction of their plant would be postponed. USA based Protein Evolution Inc. can also enzymatically hydrolyse polyester with their Biopure™ process.^133^ Former Swiss Rheiazymes performed enzymatic hydrolysis to recycle polyamide and elastane yarn.^134^ The company announced that they had to enter a liquidation process by the end of 2024.

The concentrated acid hydrolysis technology is more robust against feedstock variation when using post-consumer waste textile as the process is not hindered by the dyes. Enzymatic hydrolysis is less suitable when processing actual post-consumer waste textile as the textile waste contains a mixture of many dyes which are known for their potential to inhibit hydrolysis.^15,99,125^ Therefore, extensive pretreatment is required to minimise the inhibiting effect of the dyes on the enzymes.

Nevertheless, the high selectivity of the enzymes does allow for stepwise recycling of multiple component materials such as polyester/cotton/wool.^135^ Recycling of polyester/cotton/wool textiles will be less feasible with acid hydrolysis, as the wool will get (partially) hydrolysed by the acid, resulting in a contamination of the acid with amino acids, requiring an acid cleaning during recycle.

### 4.2 Sustainability

Whereas homogeneous textiles are more suitable for direct fibre-to-fibre recycling, the current best option for blended textiles remains downcycling. With the suitable polycotton valorisation methods discussed earlier, the environmental burden of polycotton waste textile could be reduced substantially. The valorisation of polycotton waste aligns with SDG 12; Responsible Consumption and Production. However, as SDG 9 states, one should promote sustainable industrialisation. Therefore, recycling technologies should be designed in such a way that its environmental impact is minimised.

Initial results showed that concentrated acid hydrolysis with 43 wt% HCl,^57^ and enzymatic hydrolysis with a concentrated phosphoric acid pretreatment^99,109^ had a relatively low energy consumption combined with high glucose yields. This made these technologies more sustainable compared to the other considered studies.

However, the laboratory scale reactions operated at a solid loading of 20–50 g L^−1^. This should be optimised during the scaling of the reactor to improve the efficiency and, consequently, the sustainability of the processes as it would decrease the amount of waste produced per unit of glucose. Additionally, the materials used during textile valorisation should be recycled wherever possible to minimise the environmental impact of the process.

### 4.3 Economic viability

Any process going from lab scale to pilot scale needs to be economically viable. Enzymatic hydrolysis can yield close to 100%, however, to obtain such a high yield high enzymatic loadings, with a high enzyme cost, and extreme long reaction times are needed.^15^ Additionally, enzymatic hydrolysis can only be effective when a pretreatment is performed, which adds substantially to the production cost. Therefore, acid hydrolysis is considered a more cost-effective hydrolysis methods provided the glucose yields can be improved to at least 70–80%.^73,74^

Acid hydrolysis is most commonly performed with a low sulfuric acid concentration at a high temperature. To obtain a high glucose yield, the material also needs to be pretreated with concentrated sulfuric acid. Although such a process leads to a high glucose yield, the high temperature and two-step process lead to an enormous increase in production costs when considering large scale production. When using a one-step process where the hydrolysis is performed with a high acid concentration at a low temperature, the process is much simpler and the production costs are correspondingly lower. High concentrated acid processes, however, do require acid recovery/recycling and acid-resistant vessels.^69^ At ambient temperature and pressure, commercially attractive plastics such as poly(vinyl chloride) can be used as material of construction.^57^

### 4.4 Safety

Acid hydrolysis, concentrated acid hydrolysis in specific, comes with several safety concerns. Mineral acids such as sulfuric acid, hydrochloric acid and phosphoric acid are highly corrosive, requiring specialised equipment. In addition, the highly concentrated acids can release harmful fumes. For dilute acid hydrolysis, on the other hand, high temperatures are needed which require additional safety precautions as well. Enzymatic hydrolysis itself is considered safer than acid hydrolysis as the conditions are less severe. However, the pretreatments needed when performing enzymatic hydrolysis often include the use of concentrated acids or other chemicals which need to be handled with care. Thus, depending on the pretreatment needed, enzymatic hydrolysis can have fewer safety concerns compared to acid hydrolysis.

## 5 Conclusion

The world's ambition to move away from fossil resources has led to a significant increase in interest in the valorisation of textile waste. Especially polycotton waste is of interest, as the blended material is difficult to recycle. Cotton-containing textiles are particularly interesting as these could produce an alternative glucose stream for the chemical industry, which is not in competition with the food industry. To fully valorise the polycotton waste textile, complete separation of both fractions is wanted. Additionally, solid PET residue is desired so the polyester can be converted into recycled polyester.

Thus, the goal of polycotton waste valorisation is (a) to produce a glucose stream that can be further used in the chemical industry and (b) to obtain solid polyester that can be used further for recycling. To enable effective cotton recycling, this review focuses on acid and enzymatic hydrolysis.

Acid hydrolysis can be divided into dilute acid and concentrated acid hydrolysis. Dilute acid hydrolysis is often paired with a concentrated acid pretreatment to provide proper solubilisation of the cellulose. Concentrated acid hydrolysis does not require a pretreatment. Enzymatic hydrolysis needs to be combined with a pretreatment as well, as the high crystallinity of cellulose reduces the enzymatic activity. Enzymatic hydrolysis of cellulose is performed with cellulases, which consists of endo-, exoglucanases and β-glucosidases. These enzymes work synergistically to hydrolyse cellulose into glucose. Due to the high selectivity of enzymes, a glucose yield up to 98% can be obtained.

When considering the yield and energy consumption of all suitable studies for polycotton valorisation, a concentrated acid hydrolysis with 43 wt% HCl at room temperature without pretreatments is the most energy efficient with a yield of 80% glucose. An enzymatic hydrolysis with an 85 wt% H_3_PO_4_ pretreatment showed to be the most energy efficient with a reducing sugar yield of 93–99%.

Although significant laboratory research has been done on the hydrolysis of cotton to glucose, there are only a limited number of processes that have entered the pilot plant scale. Currently, both an acid hydrolysis and an enzymatic hydrolysis process are at a TRL of 6, indicating that both technologies have been tested in relevant environments. However, further research is needed to generate a safe and technological feasible process which can operate in a sustainable way.

This review showed that using textile waste as alternative feedstock for the current chemical industry has potential and needs further research so the enormous quantities of textile waste can be tackled and the chemical industry can be supplied with a more sustainable feedstock.

## Author contributions

Nienke Leenders: writing the original draft, review and editing. Gerard P. M. van Klink: supervision and review. Gert-Jan M. Gruter: supervision and review.

## Conflicts of interest

There are no conflicts to declare.

## Supplementary Material

SU-003-D5SU00230C-s001

## Data Availability

No primary research results, software or code have been included and no new data were generated or analysed as part of this review.
