# Supplementary material for: Towards polycotton waste valorisation: depolymerisation of cotton to glucose with polyester preservation
Source: RSC Sustain. 2025 Jul 24;3(9):3863–82. doi: 10.1039/d5su00230c (PMC12287909; doi:10.1039/d5su00230c)
Supplement: SU-003-D5SU00230C-s001 [file SU-003-D5SU00230C-s001.pdf]

Supplementary Information (SI) for RSC Sustainability.
This journal is © The Royal Society of Chemistry 2025

Supplementary Information

Towards polycotton waste valorisation: Depolymerisation of cotton to glucose with polyester preservation

Nienke Leenders, [a] Gerard P. M. van Klink [a,b] and Gert-Jan M. Gruter [a,b*]

Affiliations

[a] *Van 't Hoff Institute for Molecular Sciences, University of Amsterdam, Science Park 904, 1090 GD Amsterdam (the Netherlands)*

[b] *Avantium Support BV, Zekeringstraat 29, 1014 BV Amsterdam (the Netherlands)*

*Corresponding author. E-mail address: g.j.m.gruter@uva.nl

1. Cotton production process

1.1 From plant to fabric

1.1 Fibre production

Cotton is the most used natural fibre in the textile industry due to its unique properties such as durability, water absorbance and comfortable wear. Cotton, a staple fibre, is mainly produced from the *Gossypium hirsutum* (90% of the cotton production).[1,2] Each cotton plant has several cotton bolls, that contain long fibres (lint, length = 22–32 mm), suitable for the textile industry, and short fibres (linters/fuzz fibres, length = 1.5–10 mm), used for coarse fibres or other cellulose products such as paper. The cotton bolls are harvested whereafter the fibres are separated from the seed in the ginning process. The ginning process consists of separating the lint from the linters, seed and plant foreign material and includes the baling of the fibres.[1,3] The raw cotton after ginning contains approximately 95% cellulose and 5% noncellulosic material such as waxes, pectins and proteins from the cuticle and primary wall.[1,4] The quality of the fibre is affected by the geographical climate parameters, harvesting method, non-cotton and moisture content and the ginning method.[3]

1.2. Yarn production

Before the cotton fibres can be used for the production of apparel, they need to be converted into yarns and fabrics. The production of yarn from raw cotton fibres requires numerous steps (Fig. 1), which can be categorized in the following sections: opening and cleaning, blending, drafting and spinning.

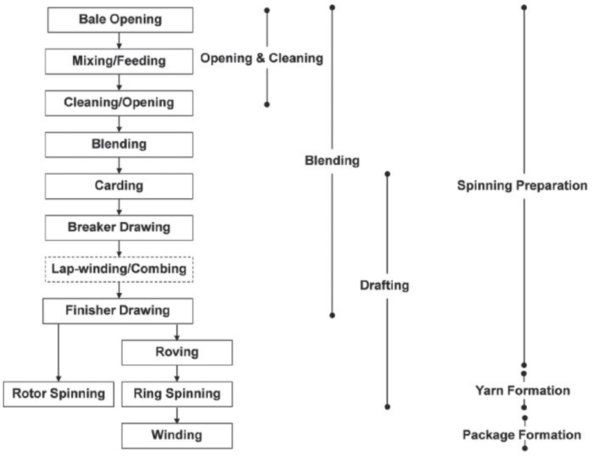

Fig. 1: Production steps needed for the production from ginned cotton to cotton yarn. Reproduced from Kelly et al. (2015). [5] Copyright (2015) with permission from John Wiley and Sons.

Opening and cleaning

After the ginned cotton fibres are baled, they are transported to the spinning mill. The bales often contain impurities such as foreign matter and gin motes. Additionally, the quality of the fibres varies within a single and between different bales. Therefore, the first steps include the opening and cleaning of the bales.

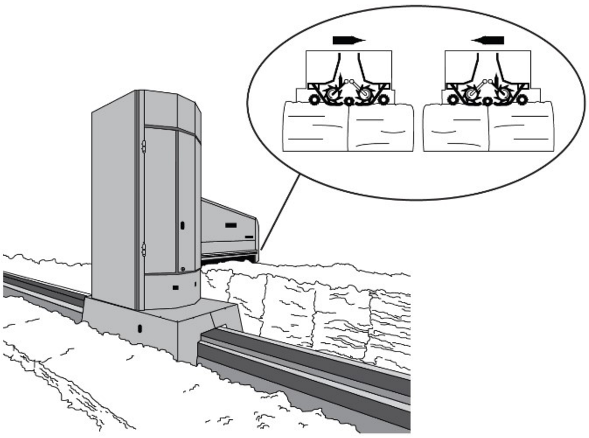

Fig. 2: Schematic representation of a bale plucker. The bale plucker removes the top layer of the bales in the laydown. From here, the cotton tufts are fed into the opening line, the nonfibrous components are removed and the tufts are opened. [6] Copyright (2001) with permission from Texas Tech University.

Blending

To obtain a homogeneous yarn strand, it is important to properly mix the bales. The first stage of blending occurs in a laydown, a long line of different cotton bales. When limited information is available about the bales' properties, a large number of bales is used. When detailed information about the properties of the fibres in the bales is available, fewer bales can be used to maintain an uniform processing performance.[5] Fiber properties considered include length, strength, uniformity, yellowness and micronaire (a measure of a fibre's maturity and fineness).[7] After arranging the bales in the laydown, the bags and ties are removed and the bales are left to 'bloom' for 24 h, as the bales are extremely dense from packing. After blooming, the bale plucker moves back and forth over the laydown, removing a small top layer of the bale which is fed to the opening line (Fig. 2).[6] As the removed cotton tufts still contain nonfibrous components, the fibres are cleaned by a series of machines that remove the nonfibrous materials and opens the tufts of fibres into individual fibres.

After cleaning and opening of the tufts, the fibres are handled in the carding process (Fig.3). Carding further removes fine debris to obtain an acceptable level of contaminants.[5,8] Additionally, the fibres are all aligned in a parallel direction with a minimal mass variation. After carding, a sliver of cotton fibres is obtained for further processing.

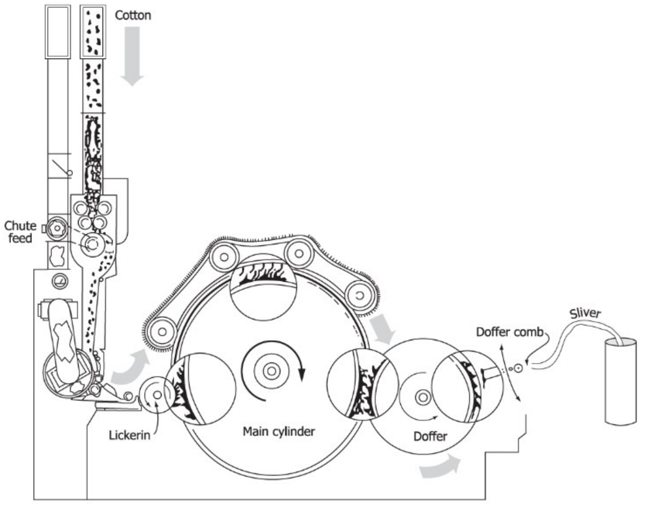

Fig. 3: Schematic representation of the carding machine. The carding machine cleans and aligns the fibre into a sliver. [6] Copyright (2001) with permission from Texas Tech University.

Drafting

Carding is followed by breaker drawing of the slivers. Drawing straightens the fibres in the sliver, combines several slivers into one and produces a more uniform sliver.[8] This process uses drafting rollers, where the thickness of the sliver is controlled by the different speeds of paired rollers (Fig. 4).[5] As the sliver passes through these rollers, the surface speed increases with each subsequent set. After drawing, the fibres in the sliver are nearly straight and parallel to the sliver's axis.

Drawing can be followed by combing of the sliver. Combing removes short fibres, hooks from long fibres and any neps (small knots) from the cotton, resulting in a clean and lustrous sliver.[5] Before yarn formation, the slivers are drawn for a second time in the finisher drawing, using the same kind of machine as the breaker drawing. This step produces a sliver of the desired size suitable for yarn production.

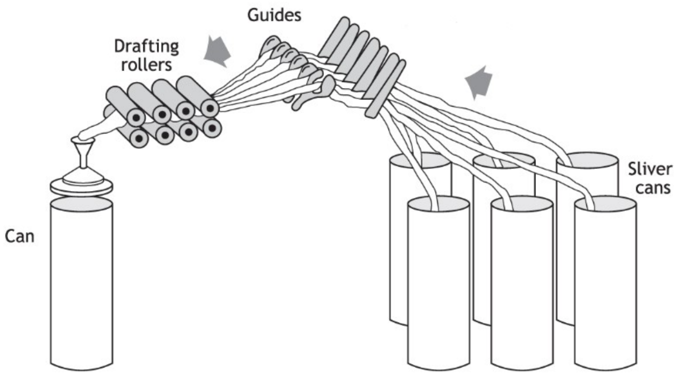

Fig. 4: Schematic representation of the drawing process. Drawing straightens the fibres, blends several slivers and produces one more uniform sliver. [6] Copyright (2001) with permission from Texas Tech University.

Spinning

Approximately 95% of all yarn is manufactured using rotor and ring spinning methods, with rotor spinning contributing to 25% of the overall production.[5] During spinning, the fibres are converted into yarn by twisting of the fibres. In rotor spinning, the sliver is fed into the system with the feed roll, where the comber roll plucks the fibres from the sliver and guides them into the spinning rotor (Fig. 5). The fibres are transported to the outer edge of the rotor by air currents and centrifugal force while being evenly distributed. A starter yarn continuously draws the yarn from the centre of the rotor. Thereafter, the yarn is wound on a spindle and ready for fabric manufacturing.

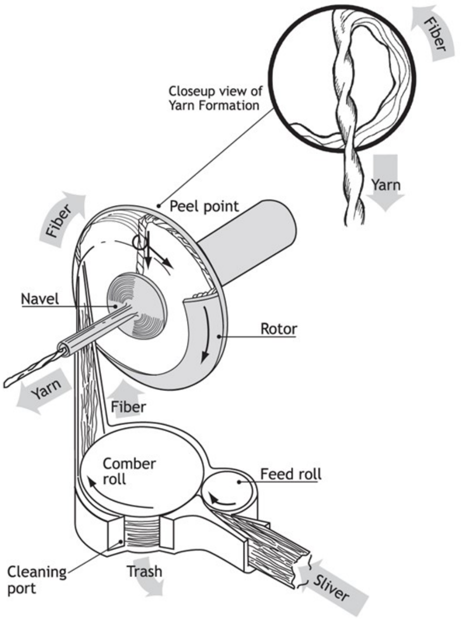

Fig. 5: Schematic representation of an open end rotor spinner. The sliver enters the spinner via the feed roll where the comber roll plucks the fibres from the sliver and guides them into the spinning rotor. The yarn is produced due to the convolutions of the rotor.[6] Copyright (2001) with permission from Texas Tech University.

Before slivers can be processed via ring spinning, they need to be roved. Roving produces a fibre package, which can be conveniently used in the ring-spinning machine. Roving significantly reduces the size of the sliver and twists the sliver.[5] The roving process involves three sets of rollers, with the surface speed of the rollers increasing with each subsequent roller set (Fig. 6). The roving is wound onto the roving bobbin, which is then transferred to the ring spinner.

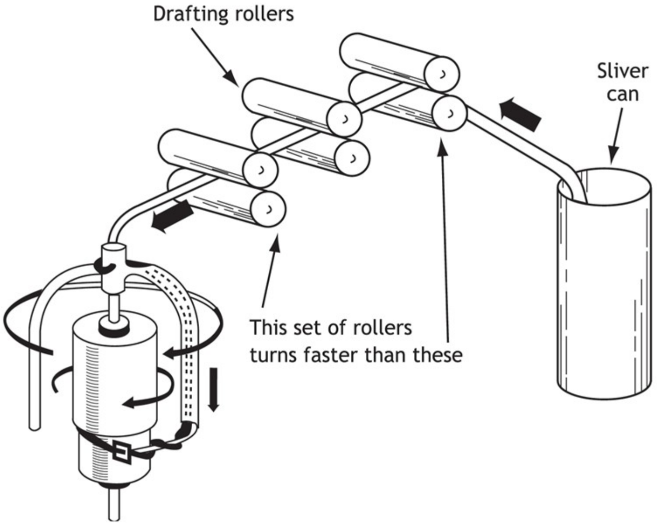

Fig. 6: Schematic representation of the roving process. Roving uses drafting rollers and a bobbin to produce a convenient fibre package for the ring-spinning process.[6] Copyright (2001) with permission from Texas Tech University.

The majority of the yarn (75%) is spun via ring spinning, predominantly used for the production of fine yarns.[5] During ring spinning, the desired size and twist of the yarn is obtained. From the roving bobbin, the roving is first further drafted in the drafting system whereafter it is twisted and wound onto the bobbin with the ring and traveller (Fig. 8). Modern ring-spinning machines typically have spindles that rotate at speeds ranging from 15,000 to 20,000 revolutions per minute. The thickness of the yarn is measured in tex (grams per 1000 meter) or denier (den) (grams per 9000 meter), where a higher tex or den indicate a thicker yarn.

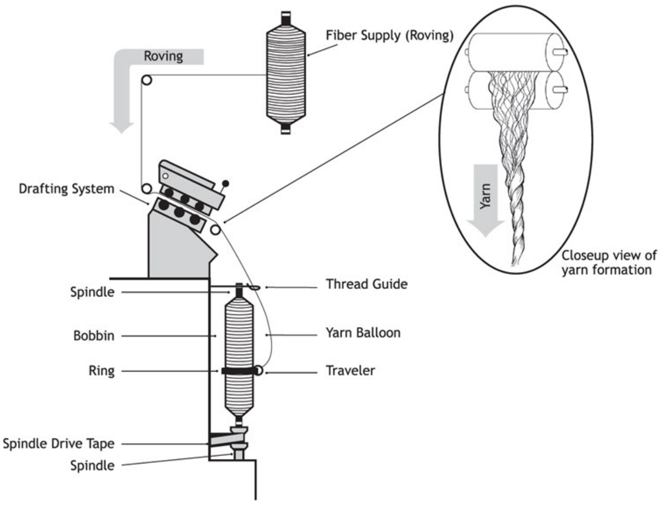

Fig. 7: Schematic representation of the ring spinning process. The roving is further drafted whereafter the sliver is rotated into yarn. The yarn is spun on a bobbin with the traveler.[6] Copyright (2001) with permission from Texas Tech University.

1.2 Fabric production

Fabrics are produced from yarn through either weaving or knitting. In the weaving process, lengthwise (warp) yarns and crosswise (weft) yarn are interlaced.[5] Weaving is performed on a loom, where the warp yarn is raised and lowered by the heddle connected to the harness (Fig. 8). After each change in position, a weft yarn is inserted and tightened with the reed. The harnesses then change position and another weft yarn is inserted. The produced fabric is rolled on a cloth beam.

Fabrics can also be formed through knitting. Knitting forms loops with a single continous yarn and joins the loop with the neighboring loop to form the fabric. This can be done using latch needles with a hook and latch mechanism or by a spring device. Wales are the loops that form a series of chains along the length of the fabric, while courses are loops across the width of the fabric. Wales and courses in knitted fabrics can be compared to the warp and weft in woven fabrics. However, a knitted fabric can either be a warp knit or a weft knit. Warp knits have the yarn moving lengthwise of the fabric, whereas weft knits have the yarn moving crosswise. Knitting machines often use wax or lubricant to promote the movement of the yarn and reduce fly.[5]

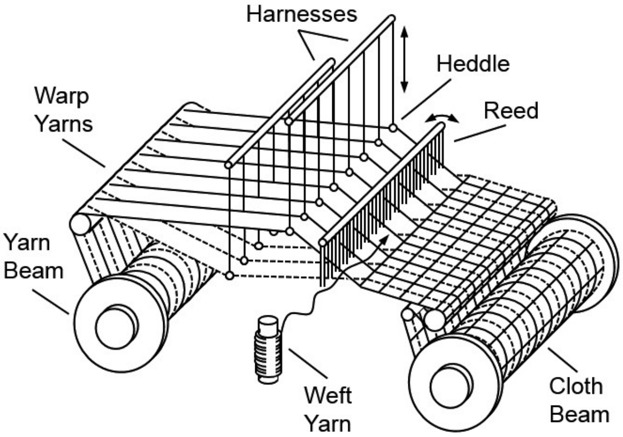

Fig. 8: Schematic representation of a loom. The heddle and harnesses are use to move the warp yarn, whereafter the weft yarn can be inserted and tightened with the reed. Reproduced from Kelly et al. (2015).[5] Copyright (2015) with permission from Wiley and Sons.

1.3 Modification of cotton fabric

After the yarn is produced, it can be subjected to several preparation steps in the following order to improve its processability and appearance: sizing and desizing, scouring, bleaching, mercerization, dyeing and finishing.

Sizes are adhesive chemicals such as starch, carboxymethyl cellulose, carboxymethyl starch and polyvinyl alcohol, which increase the mechanical strength of the yarns during weaving to prevent breakage.[9] The sizing agents are applied before the weaving and need to be removed afterwards as sizing agents can reduce the dyeability of the fabric.

After desizing, the fabric is subjected to scouring. A mature cotton fibre has a noncellulosic covering, the cuticle, which contains waxes, pectins and proteins left from biosynthesis. Removing these impurities from the fibre allows for proper finishing and dyeing of the fibre.[9] After removal of the impurities with a sodium hydroxide solution, the cellulose content is over 99 wt%. To remove any coloured impurities, the material can be bleached.[9]

Mercerization is another important industrial treatment where cotton is swollen with an aqueous sodium hydroxide (NaOH) solution. Mercerization improves the dye affinity, chemical reactivity, dimensional stability, tensile strength, lustre and smoothness of the cotton fabric.[10,11] During mercerization, the crystal structure of cellulose is irreversibly converted from cellulose I into cellulose II.[12] Mercerization is performed at a NaOH concentration of 5–8 wt%, low temperature (0–20 °C) and with a relatively short contact time (seconds to minutes) under tension.[1,12–14] The swelling causes the formation of large pores in the fibre, leading to a greater depth of colour or dye yield of the fibre.

Another important commercial treatment of cotton is the dyeing of the fabric. Dyes consist of a chromophore, auxochrome and matrix. The chromophore is responsible for the colour of the dye molecule and the auxochrome deepens the colour of the dye and can affect the solubility and binding of the dye. Cotton can be dyed with reactive, direct, vat and sulphur dyes. From these four types of dyes, only reactive dyes form covalent bonds with cotton. Direct dyes form hydrogen bonds and Van der Waals bonds and vat and sulphur dyes are physically bonded in their water insoluble form. The procedure for dyeing cotton fibres has been extensively reviewed and more information on the classification, synthesis and application of dyes can be found in the work of Benkhaya et al. (2020).[15]

Finishing is the last step in the production process of textiles. Finishes can be used to increase the softness and lustre of the fabric, make the fabric flame-retardant, antimicrobial, resistant against wrinkles and increase the ultraviolet protection ability of a fabric.[5]

2. Polyester production process

The production of PET fibres entails several steps, namely PET synthesis, spinning, drawing and winding (Fig. 9).

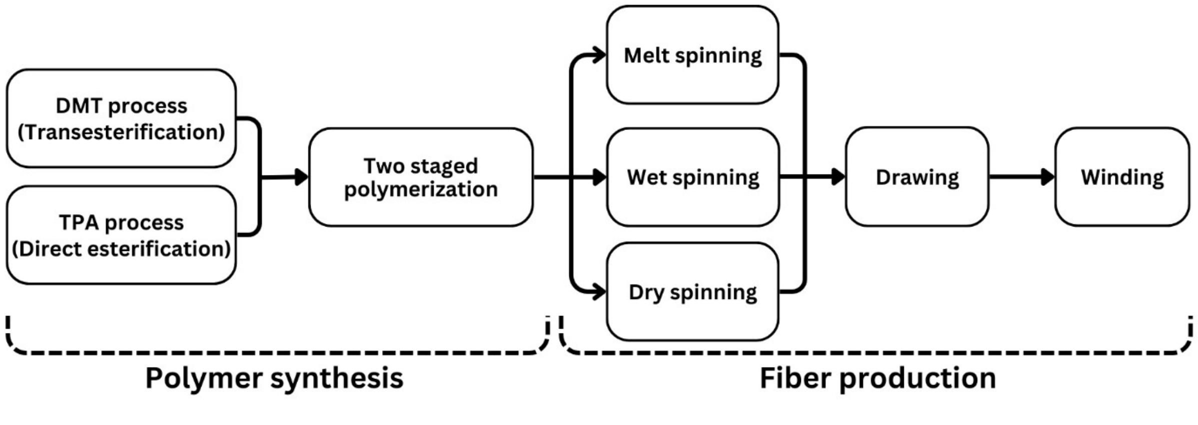

Fig. 9: Schematic overview of PET filament fibre production.

2.1 Synthesis of PET

PET is commercially synthesized through a two-step synthesis process, consisting of a pre-polymerization and a polycondensation step.[16] The pre-polymerization step syntheses bis-(2-hydroxyethyl) terephthalate (BHET), which can be produced via the transesterification process of dimethyl terephthalate (DMT), or via the direct esterification process of terephthalic acid (TPA).[17] Although other processes have been patented and commercialized, the DMT and TPA process continue to be the most important ones.[18]

In the DMT process, BHET is produced via transesterification of DMT with an excess of ethylene glycol (EG) (EG/DMT>1) between 150–200 °C at 100 kPa in the presence of a homogeneous catalyst for 3– 6 h (Scheme 1).[17,19,20] The catalyst is often

a metal oxide, metal acetate or mixture of both.[20] Since the reaction is reversible, constant removal of the methanol co-product is required to obtain high conversion of the methyl ester groups.[21]

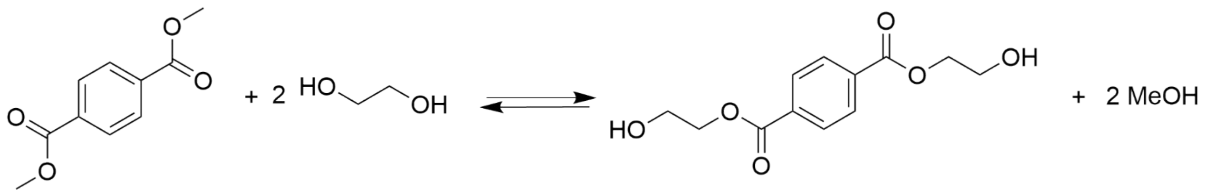

Scheme 1: Synthesis of bis(2-hydroxyethyl)terephthalate from dimethyl terephthalate and ethylene glycol via the transesterification process.

The TPA direct esterification process starts with of TPA and EG (Scheme 2). The reaction is performed by gradually increasing the reaction temperature from 250 to 290 °C for 3 to 6 h under pressure (400 kPa) in the presence of a metal oxides catalyst while simultaneously removing water to drive the reaction to the production of BHET.[16]

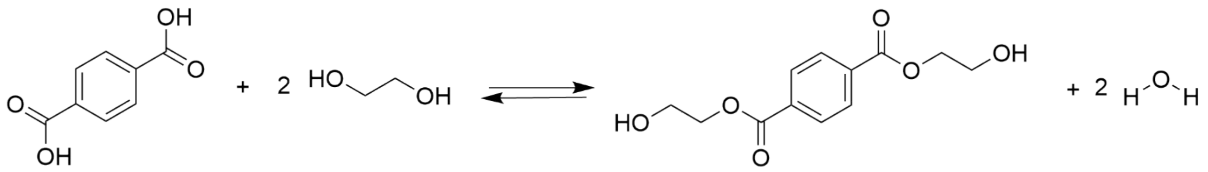

Scheme 2: Synthesis of bis(2-hydroxyethyl)terephthalate from terephthalic acid and ethylene glycol via the direct esterification process.

The BHET produced from either the DMT or the TPA process is polymerized to PET in a two-staged polymerization reaction starting at high temperature (250–300 °C) under reduced pressure (~4 kPa), followed by a further condensation process at (slightly higher) temperature (280–300 °C) under more reduced pressure vacuum (<0.13 kPa) with an antimony trioxide catalyst while continuously removing the EG formed (Scheme 3).[17,21–24] Overall, the PET produced via the DMT process is less stable compared to the TPA process as the PET fibre contain more catalyst residue which remain catalytically active.[18]

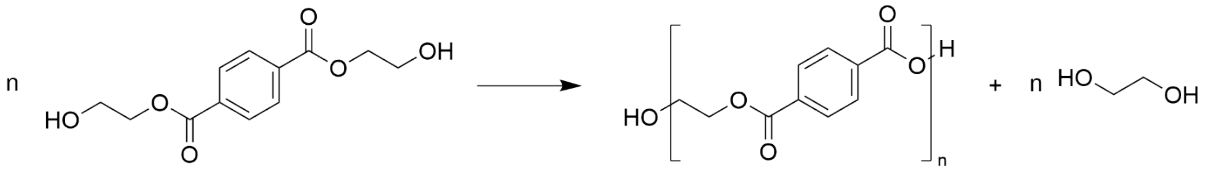

Scheme 3: Synthesis of poly(ethylene terephthalate) from bis(2-hydroxyethyl)terephthalate via polycondensation.

2.2 PET fibre production

After polymerization, PET fibres are predominately produced via melt spinning, wet spinning and dry spinning. Melt spinning, the most common method for PET, uses the molten polymer (dope fluid) from the polymerization process which is directly pumped through the spinneret. The spinning occurs at a temperature of 280–300 °C, and a pressure of 100–200 bar is required to push the polymer through a filtration media to remove any impurities, whereafter it enters the spinneret. The viscoelastic filaments exiting the spinneret are solidified via cooling with air.

With wet and dry spinning, polymer granulates are melted to obtain the dope fluid whereafter it is pushed through the spinneret. When using polymer granulates, it is very important to store the granulates in a dry environment, as only 0.01% water can cause a 10% decomposition of the polymer during re-melting.[25] For wet spinning, the viscoelastic filaments are solidified by submerging them in a coagulation bath where with dry spinning they solidify in a heating chamber. Depending on the spinning conditions, this will either lead to a higher crystallinity due to a higher generated molecular order or reduce the crystallinity as an effect of the enormous increase in surface area per unit volume.[26]

Disregard of the spinning processes used, all processes are sequenced with a drawing step to generate molecular order and orientation, elongate the fibres and decrease the diameter.[18] Drawing is performed by stretching the fibres between rollers, which can be done in the presence or absence of a heating source. When heat is applied, the temperature should be above the glass transition temperature. Thereafter, the fibre is wound around a bobbin and can be used for the production of yarn.

The eventual fibre structure, and thus properties, depends on the processing temperature, rate of stretching, spinneret size, draw ratio, heat conditions and relaxation of the polymer.

As melt spinning occurs at temperatures above the melting point, the material can degrade. One of the main degradation products is the production of diethylene glycol (DEG) in the backbone, which reduces the crystallinity and decreases the hydrolytic and thermal stability. The DEG quantity in PET produced in the direct esterification of TPA is higher compared to the quantity in PET from transesterification of DMT.[27] Therefore, a heat treatment is often applied to remove the DEG rich portion from the amorphous region.

3. Energy consumption calculations

For the energy consumption calculations, the following formulas are used and assumptions are made.

$$Q = mc\Delta T$$

where Q = Heat (J), m = mass (kg), c = specific heat capacity (J/kg °C) and ΔT = difference in temperature between the reaction temperature and reference temperature (room temperature (25 °C).

The energy consumption is based on the energy needed during the pretreatment and hydrolysis. The calculations consist of the heat needed to reach the reaction temperature and the heat needed to stay at the reaction temperature during the entire reaction time. The following assumptions are made:

1. The mass for all calculations is 1 kg, linked to a 1 litre scale.
2. The specific heat capacity for all calculations is assumed to be that of water (4184 J/kg*C), except for experiments using >80% sulfuric acid (1465 J/kg*C), >81% Phosphoric acid (1859 J/kg*C), Formic acid (2201 J/kg*C), 50-60% sulfuric acid (2428 J/kg*C).
3. Heating the reaction takes 5 minutes to get the reaction at the desired reaction temperature.
4. The heat loss of the reactor is 1.1 W.
5. When no reaction temperature for enzymatic hydrolysis was noted in the article, 50C was used.
6. For overnight reaction, 12 hours is calculated
7. The energy use for mechanical refining is neglected as Vera et al. 2023 state that the energy use is 0.18kW/ton and only 50 gram is needed for 1 litre scale.

References

1 P. J. Wakelyn, N. R. Bertoniere, A. D. French, D. P. Thibodeaux, B. A. Triplett, M.-A. Rousselle, W. R. Goynes, J. V. Edwards, L. Hunter, D. D. Mcalister and G. R. Gamble, in *Handbook of Fiber Chemistry*, ed. M. Lewin, 2006, pp. 521–670.

2 D. D. Fang, in *Cotton Fiber: Physics, Chemistry and Biology*, ed. D. D. Fang, Springer International Publishing, Cham, 2018, pp. 1–11.

3 M. Dadgar, in *Cotton Science and Processing Technology*, eds. H. Wang and H. Memon, Springer, 2020, pp. 61–78.

4 Y. Liu, in *Cotton Fiber: Physics, Chemistry and Biology*, 2018, pp. 75–94.

5 B. Kelly, N. Abidi, D. Ethridge and E. F. Hequet, in *Cotton*, Wiley Blackwell, 2015, vol. 57, pp. 665–744.

6 R. W. Seagull, *Cotton Fiber Development and Processing: An illustrated overview*, International Textile Center, Texas Tech University, Lubbock, Texas, 2001.

7 G. R. Gamble, *J Cotton Sci*, 2007, **11**, 98–103.

8 N. Bakhsh, M. Qamar Khan, A. Ahmad and T. Hassan, in *Cotton Science and Processing Technology*, eds. H. Wang and H. Memon, Springer, 2020, pp. 143–164.

9 G. Varadarajan and P. Venkatachalam, *Environ Chem Lett*, 2016, **14**, 113–122.

10 P. Mansikkamäki, M. Lahtinen and K. Rissanen, *Cellulose*, 2005, **12**, 233–242.

11 X. Colom and F. Carrillo, *Eur Polym J*, 2002, **38**, 2225–2230.

12 M. Ferro, A. Mannu, W. Panzeri, C. H. J. Theeuwen and A. Mele, *Polymers (Basel)*, 2020, **12**, 1559.

13 H. Wang, A. Farooq and H. Memon, *Wood and Fiber Science*, 2020, **52**, 13–27.

14 E. Jin, J. Guo, F. Yang, Y. Zhu, J. Song, Y. Jin and O. J. Rojas, *Carbohydr Polym*, 2016, **143**, 327–335.

15 S. Benkhaya, S. M' rabet and A. El Harfi, *Inorg Chem Commun*, 2020, **115**, 107891.

16 T. Rieckmann and S. Völker, in *Modern Polyesters: Chemistry and Technology of Polyesters and Copolyesters*, 2004, pp. 29–115.

17 M. Di Serio, R. Tesser, A. Ferrara and E. Santacesaria, *J Mol Catal A Chem*, 2004, **212**, 251–257.

18 J. Militky, *The chemistry, manufacture and tensile behaviour of polyester fibers*, 2009.

19 M. Di Serio, R. Tesser, F. Trulli and E. Santacesaria, *J Appl Polym Sci*, 1996, **62**, 409–415.

20 A. L. Jadhav, R. S. Malkar and G. D. Yadav, *ACS Omega*, 2020, **5**, 2088–2096.

21 J.-M. Besnoin, G. D. Lei and K. Y. Choi, *AIChE Journal*, 1989, **35**, 1445–1456.

22 J. H. Kim, S. Y. Lee, J. H. Park, W. S. Lyoo and S. K. Noh, *J Appl Polym Sci*, 2000, **77**, 693–698.

23 K. Tomita, *Polymer (Guildf)*, 1973, **14**, 50–54.

24 B. Duh, *Polymer (Guildf)*, 2002, **43**, 3147–3154.

25 F. Fourné, *Synthetic Fibers Machines and Equipment, Manufacture, Properties*, Hanser Publishers, Munich, 1999.

26 C. Jinan, T. Kikutani, A. Takaku and J. Shimizu, *J Appl Polym Sci*, 1989, **37**, 2683–2697.

27 S. G. Hovenkamp and J. P. Munting, *J Polym Sci A1*, 1970, **8**, 679–682.
